# Phenotypic, metabolic, and biogenesis properties of human stem cell-derived cerebellar spheroids

**DOI:** 10.1038/s41598-022-16970-1

**Published:** 2022-07-27

**Authors:** Timothy Hua, Chang Liu, Sonia Kiran, Kelly Gray, Sunghoon Jung, David G. Meckes, Yan Li, Qing-Xiang Amy Sang

**Affiliations:** 1 Department of Chemistry and Biochemistry, Florida State University, 102 Varsity Way, Tallahassee, FL 32306 USA; 2 Department of Chemical and Biomedical Engineering, FAMU-FSU College of Engineering, Florida State University, 2525 Pottsdamer St., Tallahassee, FL 32310 USA; 3 PBS Biotech Inc., Camarillo, CA USA; 4 Department of Biomedical Sciences, College of Medicine, Florida State University, Tallahassee, FL USA; 5 Institute of Molecular Biophysics, Florida State University, Tallahassee, FL USA

**Keywords:** Stem-cell biotechnology, Tissue engineering

## Abstract

Human cerebellum consists of high density and complexity of neurons. Thus, it is challenging to differentiate cerebellar-like organoids with similar cellular markers and function to the human brain. Our previous study showed that the combination of retinoic acid (RA), Wingless/integrated (Wnt) activator, and Sonic Hedgehog (SHH) activator promotes cerebellar differentiation from human induced pluripotent stem cells (hiPSCs). This study examined phenotypic, metabolic, and biogenesis in early cerebellar development. Cerebellum spheroids were differentiated from human iPSK3 cells. During day 7–14, RA and Wnt activator CHIR99021 were used and SHH activator purmorphamine (PMR) was added later to promote ventralization. Gene expression for early cerebellar layer markers, metabolism, and extracellular vesicle (EV) biogenesis were characterized. Zinc-induced neurotoxicity was investigated as a proof-of-concept of neurotoxicity study. Flow cytometry results showed that there was no significant difference in NEPH3, PTF1A, OLIG2, and MATH1 protein expression between RCP (RA-CHIR-PMR) versus the control condition. However, the expression of cerebellar genes for the molecular layer (*BHLE22*), the granule cell layer (*GABRB2*, *PAX6*, *TMEM266*, *KCNIP4*), the Bergmann glial cells (*QK1*, *DAO*), and the Purkinje cell layer (*ARHGEF33*, *KIT*, *MX1*, *MYH10*, *PPP1R17*, *SCGN*) was significantly higher in the RCP condition than the control. The shift in metabolic pathways toward glycolysis was observed for RCP condition. The EV biogenesis marker expression was retained. Mild zinc-induced neurotoxicity may exist when zinc exposure exceeds 1.0 µM. RCP treatment can promote specific cerebellar-like differentiation from hiPSCs indicated by gene expression of early cerebellar markers and regionally enriched genes. The higher cerebellar marker expression is accompanied by the elevated glycolysis with the retained EV biogenesis. This study should advance the understanding of biomarkers during early cerebellar development for cerebellum organoid engineering and neurotoxicity study.

## Introduction

Cerebellum locates in the hindbrain region and consists of highly dense and complex neuron networks in the human brain^1^. It is challenging to derive human brain cerebellar-like spheroids/organoids with similar biomarkers and function from human pluripotent stem cells (hPSCs), including both human induced pluripotent stem cells (hiPSCs) and human embryonic stem cells^2,3^. Regulating anterior–posterior (A-P) and dorsal–ventral (D-V) axes is the key for successful cerebellum differentiation^4^. Fibroblast growth factors (FGFs), Wnts, and retinoic acid (RA) are small molecules that regulate the A-P patterning, whereas those affecting the D–V patterning include Wnts, bone morphogenetic proteins (BMPs), and Sonic Hedgehog (SHH)^5^. CHIR99021 (CHIR) is a morphogen that activates Wnt pathway, and it modulates neuroepithelia differentiation in a dose-dependent manner. High concentrations of CHIR favors the hindbrain development^6^ as well as the caudalization factor RA. As a SHH activator, purmorphamine (PMR) promotes ventralization of the brain spheroids^7^. Cerebellum development starts from the formation of isthmic organizer stimulated by FGF2^8^, which prevents the forebrain differentiation pathway. FGF19 facilitates the self-organization of rostral hindbrain structures with a D-V polarity^5^, and the ventral identity is enhanced by PMR later. Stroma cell-derived factor (SDF) 1α treatment promotes generation of rhombic lip-like structure as well as laminated cerebellar plate structures^9^. Sequential utilization of the above small molecules successfully generates cerebellar spheroids with molecular layer, Purkinje cell layer, and granule cell layer as shown in our previous work^2^, but only a few markers representing the three layers were studied. A close scrutinization of cerebellar biomarkers, metabolic, and biogenesis properties of hPSC-derived cerebellar spheroids would advance our knowledge about early cerebellum development.

Metabolic pathways can shift during hPSC neural differentiation^10^ and adult stem cell aging and thus became an important indicator to be monitored during stem cell differentiation^11,12^. The progression from undifferentiated hPSCs, immature progenitor cells, to mature differentiated cells may shift the metabolism from glycolysis to oxidative phosphorylation (OXPHOS) pathway^13^. In glucose metabolism of hPSCs, the entry of pyruvate into the tricarboxylic acid cycle is inhibited by reduced activity of pyruvate dehydrogenase (PDH) by pyruvate dehydrogenase kinase (PDK). Pyruvate is reduced to replenish nicotinamide adenine dinucleotide to maintain glycolytic activity and pyruvate entering the mitochondria is converted to acetyl-CoA utilized for citrate production^13^. During hPSC early differentiation, a metabolic switch controlling histone acetylation produces acetyl-CoA through glycolysis which rapidly induces hPSC differentiation^14^. In human neural stem cell differentiation into motor neurons, a switch from glycolysis to OXPHOS was observed, and mitochondrial biogenesis but not mitochondrial mass was increased^15^. However, the phenotypic correlation of cerebellar differentiation of hPSCs with the metabolic status has not been well investigated.

Extracellular vesicles (EVs) especially exosomes play important roles in cell–cell communications during stem cell differentiation^16,17^. Exosomes are nanosized (< 150 nm) membrane vesicles that contain various proteins and microRNAs that regulate cell singling networks. EV biogenesis could be affected by cytoskeleton alterations, hypoxia condition, and mechanical stress, and may exhibit different activities for different cell types^18–20^. Our previous study evaluated the EV biogenesis for forebrain cortical spheroids derived from hiPSCs^21–23^. The EV biogenesis of hindbrain cerebellar spheroids derived from hiPSCs and the effect of the cellular phenotype on EV biogenesis marker expression remains unknown.

In particular, our previous study reported the modulation of Retinoid (using retinoic acid-R), Wnt (using CHIR99021-C), and Sonic Hedgehog (using purmorphamine-P) pathways to promote cerebellar spheroid differentiation from hiPSCs^2^. Going one step further, this study characterized the derived cerebellar spheroids using RCP treatment on three aspects: (1) early cerebellar biomarkers based on Human Protein Atlas; (2) metabolic pathway shift during cerebellar differentiation; and (3) EV biogenesis marker expression to elucidate paracrine signaling during cell–cell interactions. The goal of this study is to identify in-process control markers that can predict the cerebellar differentiation outcome in a quantitative way. Hindbrain cerebellum spheroids/organoids derived from this study can be used to generate assembloids with isogenic forebrain cerebral organoids^24–27^, to study the heterogeneity of microglia^28^ and blood brain barrier^29^ in different regions of human brain^30^, and to investigate the neurotoxicity such as those induced by zinc as shown in this study^31–34^.

## Results

### The protein expression of the cerebellar markers

**Figure 1:**
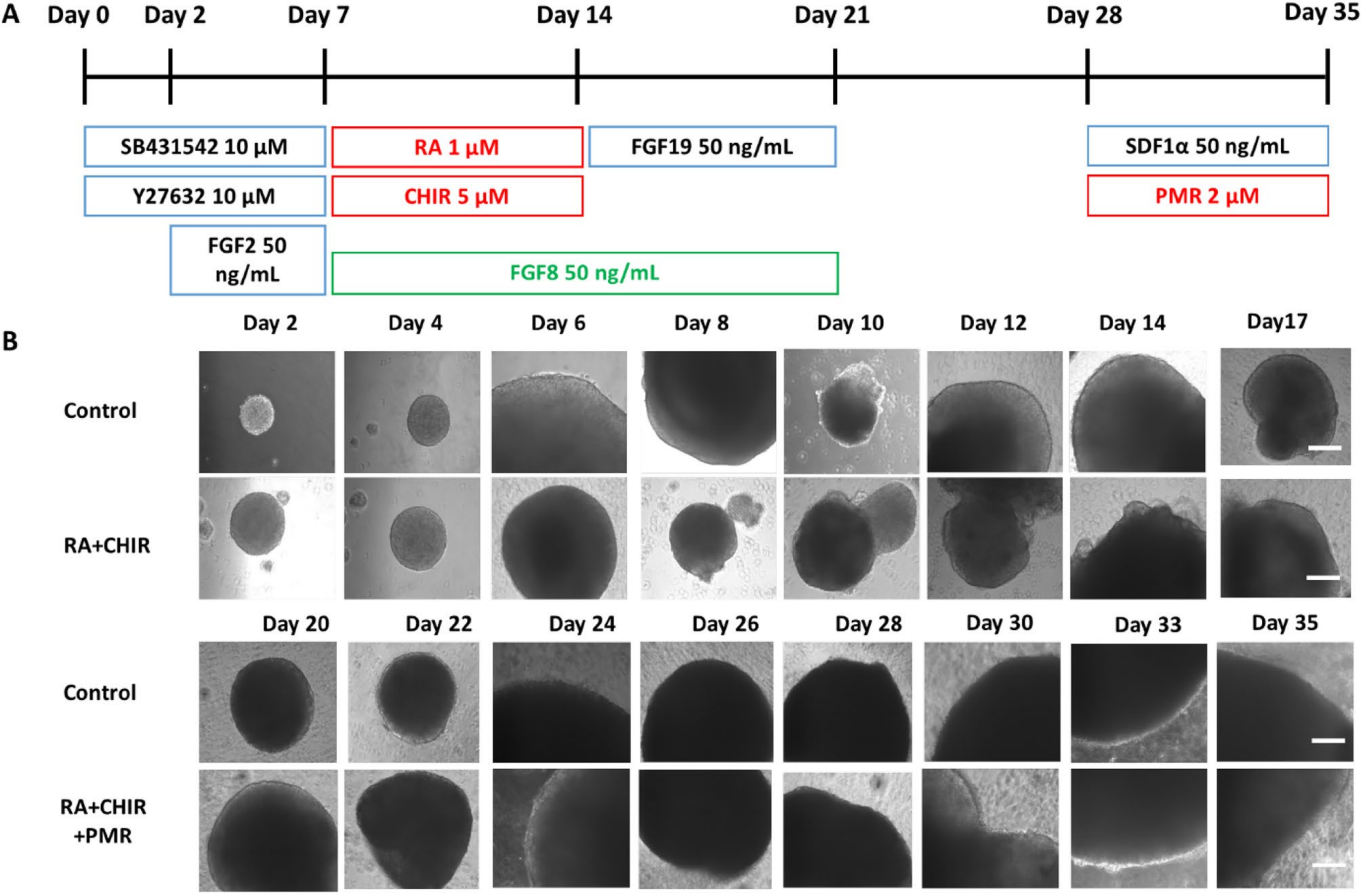
Protocols and morphology of cerebellar differentiation from hiPSCs. (**A**) Schematic illustration of the cerebellar differentiation from hiPSCs under different conditions. The control condition uses the factors written in the black font. The tested conditions are the combination of RA/CHIR/PMR (RCP) and FGF8 only. (**B**) Morphology of cerebellar spheroids during differentiation for 35 days. Scale bar: 200 µm.

**Figure 2:**
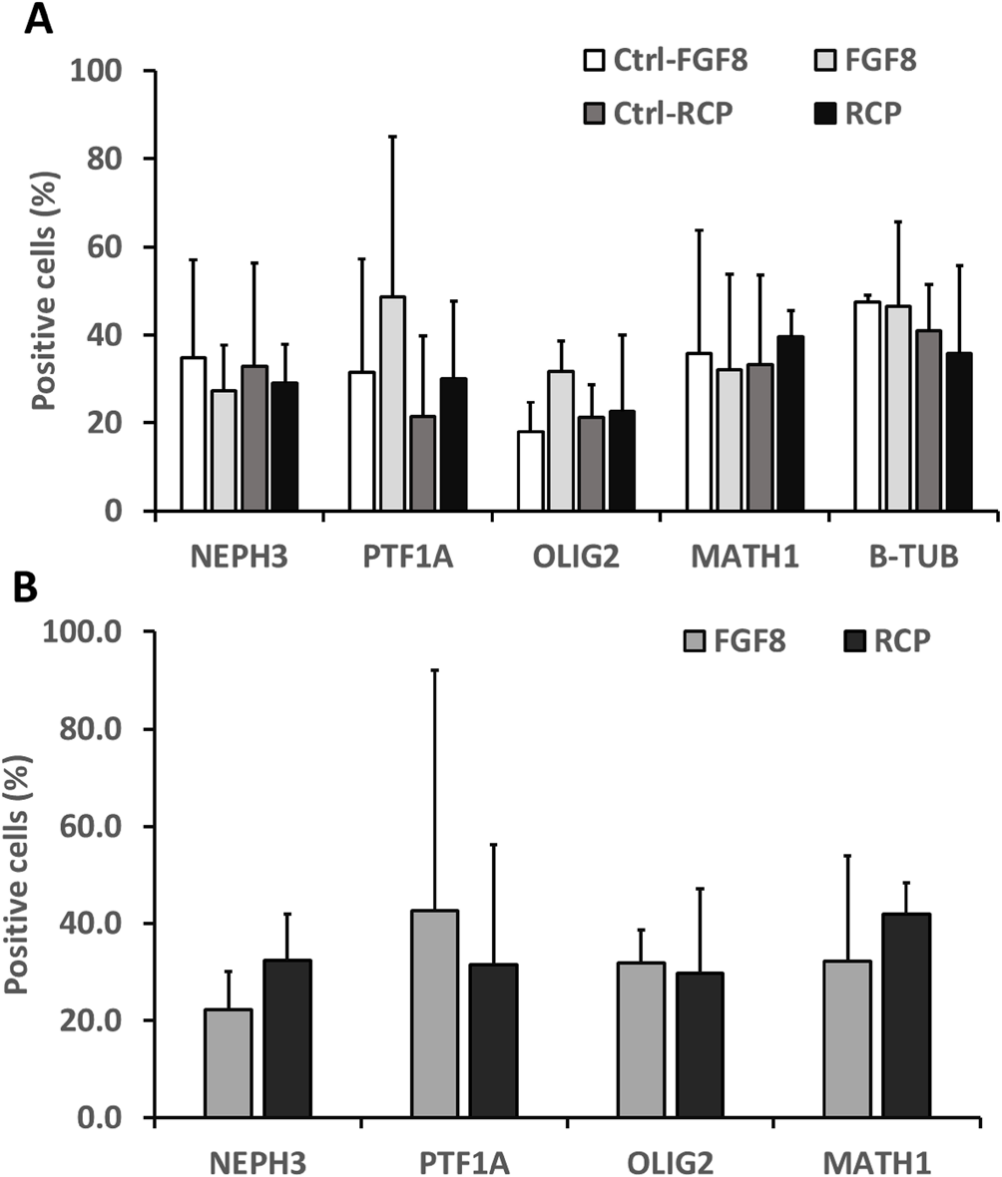
Flow cytometry analysis for the expression of cerebellar markers in the derived cerebellar spheroids. The control condition for the side-by side-comparison with RCP condition is refereed as Ctrl-RCP. The control condition for the side-by side-comparison with FGF8 condition is refereed as Ctrl-FGF8. (**A**) Day 35 RCP condition versus the side-by-side control (Ctrl-RCP) or FGF8 condition versus the side-by-side control (Ctrl-FGF8); (**B**) RCP versus FGF8 condition. The average value and the standard deviation are based on the results from three independent differentiations (each differentiation has the corresponding control condition).

The schematic illustration and morphology of cerebellar differentiation from hiPSCs are represented in Fig. 1. For the control condition, SB431542, FGF2, FGF19 and SDF1α were used as reported previously^35^. For the RCP tested condition, RA and CHIR were added on week two of culture, and PMR was added on week five. The RCP treatment can induce further caudalization of the spheroids, and therefore, it might promote hindbrain gene expression. NEPH3 and PTF1A, OLIG2, and MATH1 are chosen as the markers for the molecular layer, the Purkinje cell layer, and the granule cell layer of the cerebellar spheroids, respectively. β-tubulin III was used as the general neuronal marker. The immunostaining results, the spheroid maturation by human mesenchymal stem cells, as well as the electrophysiology results have been shown in our previous study^2^. This study focuses on the quantitative analysis such as flow cytometry and reverse transcription-quantitative polymerase chain reactions (RT-PCR) for cerebellar marker expression and the correlation with the metabolic pathway and EV biogenesis. Flow cytometry results show that there is no significant difference in protein expression of these markers between RCP versus the control or RCP versus FGF8 groups (Fig. 2A,B; Supplementary Table S1)^36^. Western blot analysis of NEPH3 and OLIG2 protein did not show significant difference between the RCP condition versus the control either (Supplementary Fig. S1). Taken together, RCP treatment does not affect cerebellar marker expression at protein levels for the common markers NEPH3, PTF1A, OLIG2, and MATH1, probably due to large experimental variations in cerebellar spheroid differentiation and flow cytometry sample preparation. In the following study, the new cerebellar marker expression, selected based on Human Protein Atlas on the cerebellum, was performed focusing on the comparison of RCP versus the control condition at a molecular level.

### The expression of new human cerebellar specific markers

**Figure 3:**
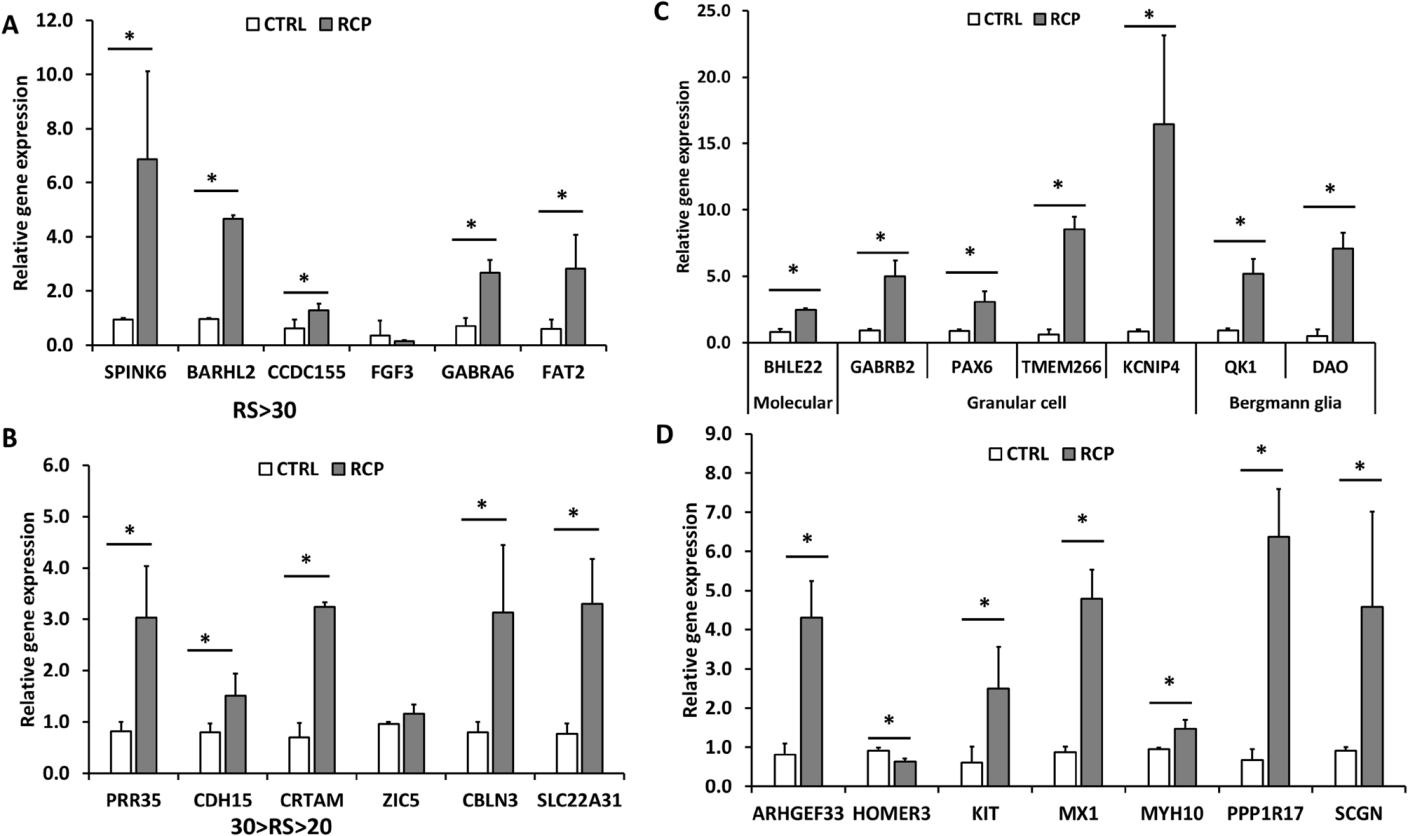
The expression of the top twelve genes with highest RS-score in the human cerebellum and cerebellar layer-specific markers. RT-PCR analysis was performed for day 35 cerebellar organoids derived from hiPSCs. (**A**) The relative expression of genes (mRNA) with RS-score greater than 30. (**B**) The relative expression of genes (mRNA) with RS-score between 30 and 20. The expression of cerebellar layer-specific markers. (**C**) The relative gene (mRNA) expression for the molecular layer, the granule cell layer, and the Bergmann glial cells; (**D**) the relative gene (mRNA) expression for the Purkinje cell layer. *p < 0.05 compared to the control condition.

According to the Human Protein Atlas^37,38^, 79% of human transcriptome are expressed in the cerebellum, and 13,188 of all genes detected in the brain are detected in cerebellum. Regionally enriched genes in the cerebellum are those that are at least four-fold higher mRNA levels in cerebellum compared to all other regions. There are 214 regionally enriched genes. In addition, regional specificity score (RS-score) corresponds to the score calculated as the fold change to the second highest region. The twelve genes with highest RS-score were selected for RT-PCR analysis in this study to compare the RCP and control conditions, including S*PINK6*, *BARHL2*, *CCDC155*, *FGF3*, *GABRA6*, and *FAT2* for RS > 30, *PRR35*, *CDH15*, *CRTAM*, *ZIC5*, *CBLN3*, and *SLC22A31* for 30 > RS > 20 (Supplementary Table S2 and S3). Our results show that the RCP condition can induce a significantly higher gene expression for most markers (10 out of 12) with the exception of FGF3 (RS-score = 38) and ZIC5 (RS-score = 24) compared to the control condition (Fig. 3A,B). The increase is ~ 2 to 7 fold for makers of RS score > 30 and 2–3 fold for markers of 30 > RS > 20. Therefore, RCP treatment can promote cerebellar differentiation from the hiPSCs at the molecular level.

**Figure 4:**
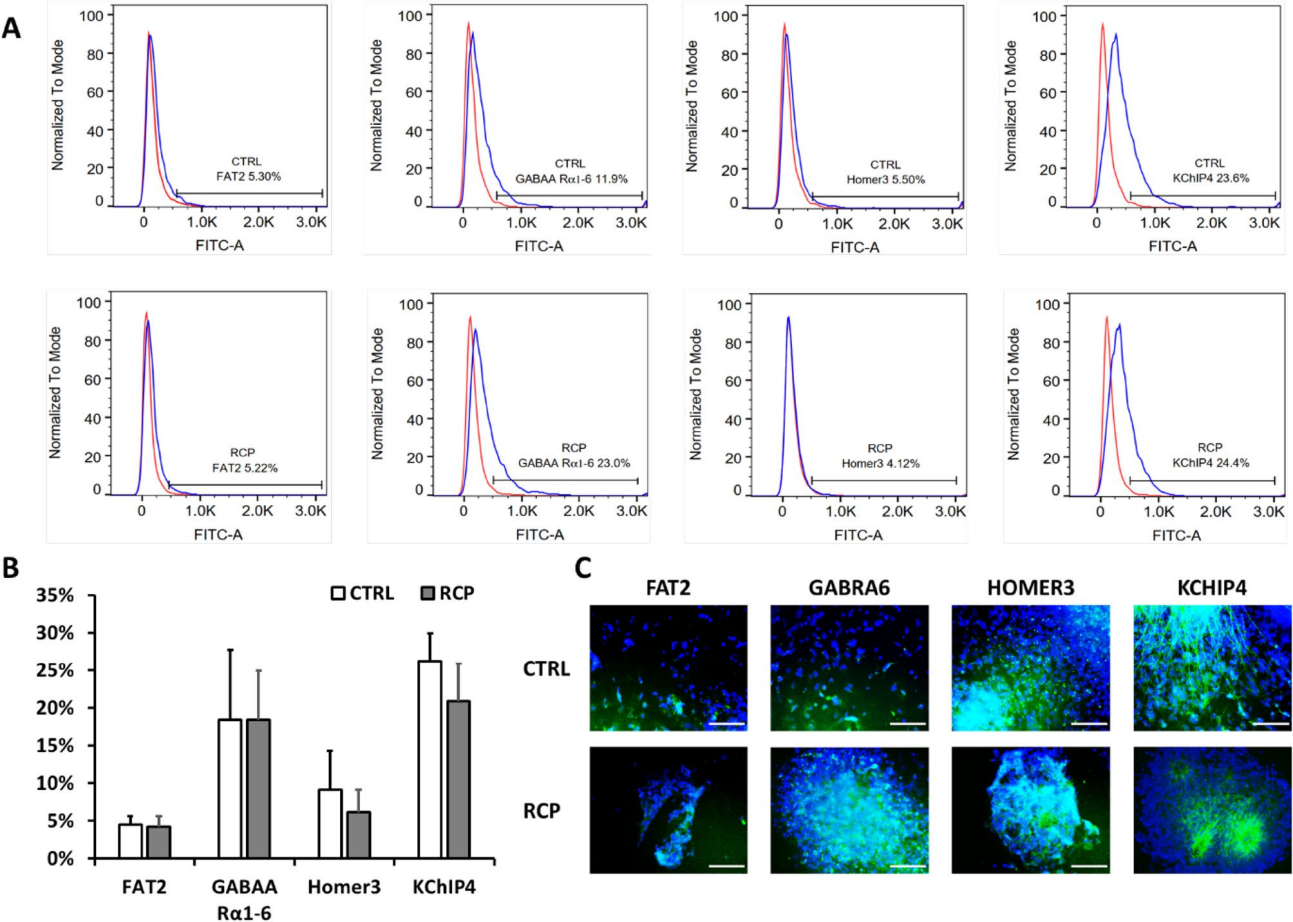
The expression of cerebellar layer-specific markers at protein levels. Four markers were selected for measuring the expression at the protein levels. (**A**) Flow cytometry histograms, red line: negative control; blue line: marker of interest. (**B**) Percentages of positive marker expression quantified by flow cytometry; (**C**) Immuno-fluorescent images. Scale bar: 100 µm. Blue: Hoechst 333342 for counterstaining with cell nuclei.

Moreover, 14 selected markers for specific cerebellar layer were examined (Supplementary Table S4 and S5). *BHLE22* was chosen as the marker for molecular layer. For granule cell layer, *GABRB2*, *PAX6*, *TMEM266*, and *KCNIP4* were examined. *QK1* and *DAO* were used as the marker for Bergmannglia cells. For Purkinje cell layer, *ARHGEF33*, *HOMER3*, *KIT*, *MX1*, *MYH10*, *PPP1R17*, and *SCGN* were determined. The expression of cerebellar specific genes for the molecular layer, the Purkinje cell layer (except HOMER3), and the granule cell layer was significantly higher (~ 2 to 15 fold) in the RCP condition compared with the control (Fig. 3C,D). Interestingly, the expression for the Bergmann glial cells, an important cell type in the cerebellum, is also higher (~ 2 to 6 fold). Therefore, RCP treatment can promote specific cerebellar layer differentiation in spheroids derived from hiPSCs at the molecular level. Selected markers were evaluated for the expression at protein levels by flow cytometry and immunocytochemistry, including FAT2, GABRA6, HOMER3, and KCNIP4 (Fig. 4). No significant difference in protein expression of these markers was observed between RCP versus the control condition.

### Metabolic pathways are altered by RCP treatment

**Figure 5:**
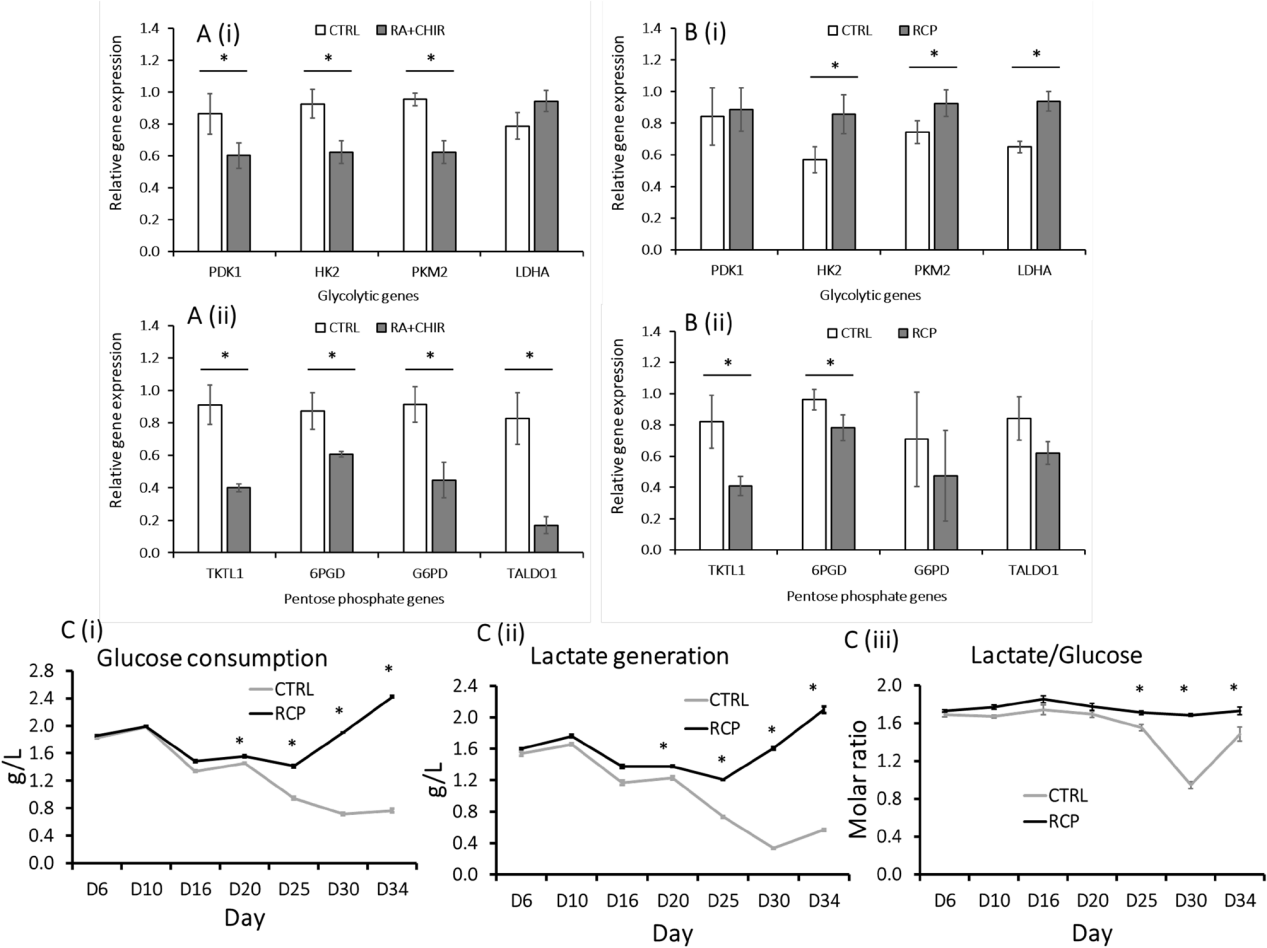
Characterizations of metabolic pathways during cerebellar differentiation of hiPSCs. RT-PCR analysis of genes (mRNA) related to glycolytic pathway and the pentose phosphate (PPP) pathway was performed for (**A**) Day 14 cells; (**B**) Day 35 cells. (i) Glycolytic pathway; (ii) PPP pathway. Spent media were collected for metabolite analysis (glucose, lactate, glutamine, and ammonia). (**C**) (i) Glucose consumption; (ii) Lactate generation; (iii) Lactate to glucose ratio (mol/mol). *p < 0.05 compared to the control condition.

To understand the cerebellar differentiation process, the status of metabolic pathways during differentiation was examined by RT-PCR for day 14 and day 35 cells (Fig. 5A,B; Supplementary Table S6). PDK1 is pyruvate dehydrogenase kinases, which shunts pyruvate away from the mitochondria. HK2 is hexokinase 2, which phosphorylates glucose to glucose 6-phosphate. PKM2 is pyruvate kinase isoform 2, which catalyzes phosphoenolpyruvate (PEP) to pyruvate. LDHA is lactate dehydrogenase A, which catalyzes inter-conversion of pyruvate and lactate. TKTL1 is transketolase-like protein 1, which enables O_2_-independent glucose degradation. 6PGD is 6-phosphogluconase dehydrogenase responsible for NADPH production. G6PD is glucose-6-phosphate dehydrogenase responsible for NADPH production. TALDO1 is transaldolase 1, which provides ribose-5-phosphate for nucleic acid synthesis and NADPH for lipid biosynthesis.

For glycolytic pathway: at day 35, glycolytic genes (*HK2*, *PKM2*, and *LDHA*) in the RCP group have higher expression levels (~ 1.5 fold) than those in control group, which is different from what was observed for day 14 (~ 1.5 fold lower for *PDK1*, *HK2*, and *PKM2*). For pentose phosphate (PPP) pathway: at both day 14 and day 35, PPP genes (*TKTL1*, *6PGD*, *G6PD*, and *TALDO1*) in the RCP group have lower expression levels than those in the control group (2–5 fold higher at day 14 for all the four markers; 1.2–2 fold for *TKTL1* and *6PGD* only). Our previous study showed that gene expression in metabolic pathways usually correlates with the metabolic enzyme function^11^. It was postulated that the RCP group has higher oxidative activity to produce more energy for activating genes involved in differentiation at day 14. At day 35, more cerebellar cells in the RCP group increase their use of glycolytic pathway to counteract oxidative stress.

Metabolite analysis (including glucose, lactate, glutamine, and ammonia as well as Na^+^, K^+^, Ca^2+^) was performed on the spent media of cerebellar differentiation (Fig. 5C; Supplementary Fig. S2). Glucose consumption and lactate generation was similar for the control an RCP conditions before day 20. After day 20, glucose consumption and lactate generation were significantly higher for the RCP condition than the control. The lactate to glucose ratio (mol/mol) was around 1.8 for the RCP condition, indicating more anaerobic metabolism (with ratio of 2.0). However, for the control condition, the ratio was lower around 1.0–1.6, especially during day 20–35, indicating more aerobic metabolism. The difference in glutamine metabolism was consistent with glucose metabolism but was shown after day 25. Na^+^, K^+^, Ca^2+^ levels were comparable for the two conditions. Together, these observations were consistent with the results from gene expression.

### RC and RCP treatment retains EV biogenesis markers

**Figure 6:**
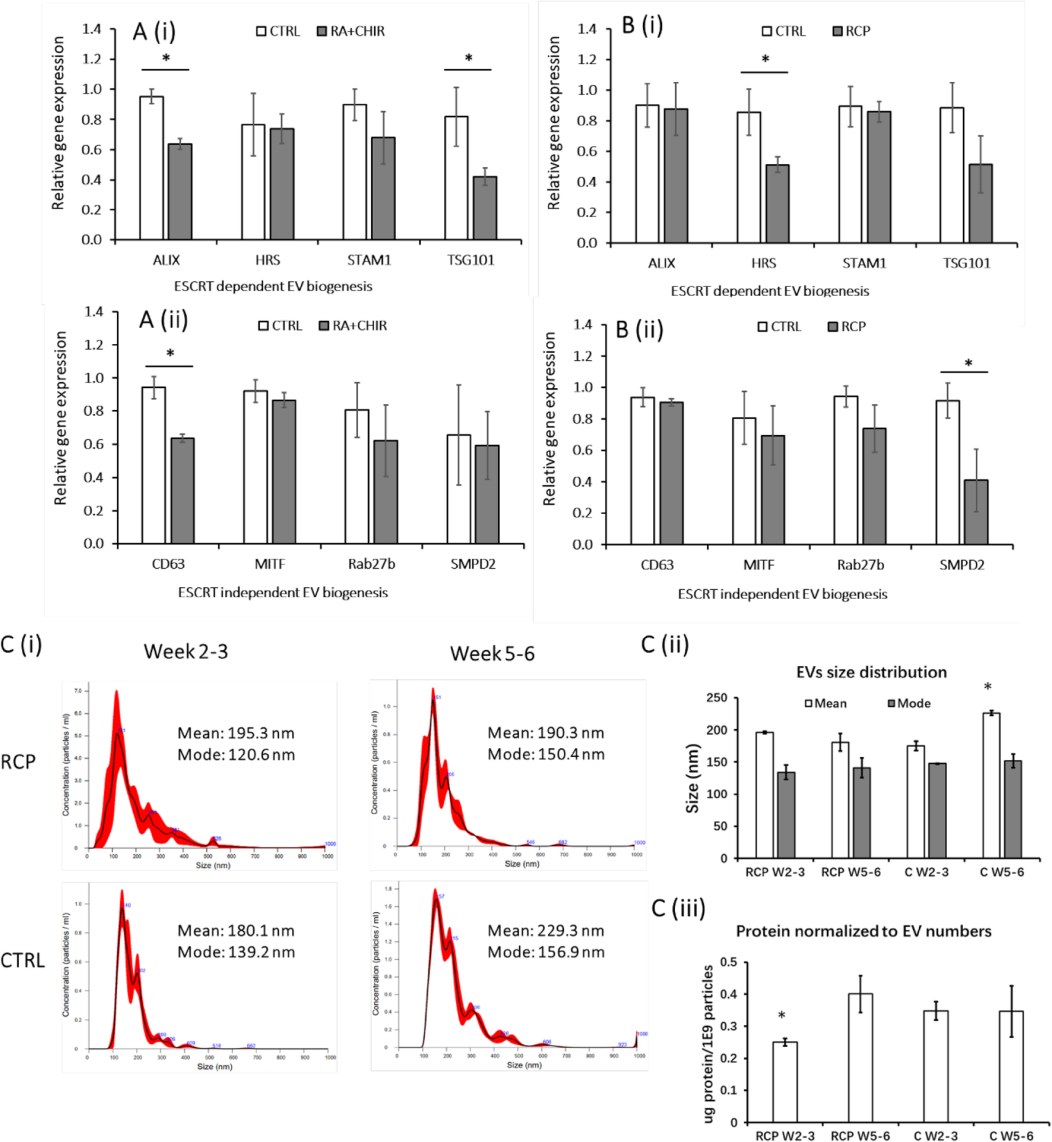
Extracellular vesicle (EV) biogenesis marker expression during cerebellar differentiation. The expression levels of EV biogenesis markers were determined by RT-PCR analysis. (**A**) Day 14 cells; (**B**) Day 35 cells. (i) ESCRT dependent: *ALIX*, *HRS*, *STAM1*, *TSG101*; (ii) ESCRT independent: *CD63. MITF*, *Rab27b*, *SMPD2*. *p < 0.05 compared to the control condition. ESCRT: the endosomal sorting complex required for transport. (**C**) (i) Extracellular vesicle (EV) size distribution determined by nanoparticle tracking analysis; EVs were isolated around day 14 (week 2–3) and around day 35 (week 5–6); (ii) Average mean and mode EV size; and (iii) protein content per 10^9^ EVs. *p < 0.05 compared to other conditions.

The EV biogenesis markers are classified as the endosomal sorting complex required for transport (ESCRT) dependent (*ALIX*, *HRS*, *STAM1*, *TSG101*) and ESCRT independent (*CD63*, *MITF*, *Rab27b*, *SMPD2*)^39^. In this study, RT-PCR analysis was performed for day 14 and day 35 cells during cerebellar spheroid differentiation (Fig. 6; Supplementary Table S7). ALIX is involved in exosome generation and cargo loading. HRS, STAM1, TSG101 are related to endosomal sorting complex required for transport. CD63 is related to exosome generation and particle packaging into exosomes. MITF is involved in exosome generation and can increase the expression of late endosomal proteins such as Rab27b. Rab27b facilitates the docking to the plasma membrane. SMPD2 facilitates the budding from the plasma membrane.

At day 14, *ALIX*, *TSG101*, and *CD63* show the decreased expression (~ 2-fold) for the RCP group compared to the control, while the other five genes show no significant difference between the control group and the RCP group. At day 35, *SMPD2* and *HRS* exhibited the decreased expression (~ 2-fold) for the RCP condition compared to the control, while the other six genes showed the similar expression. The genes having lower expression are not consistent between day 14 and day 35 cells, and they are not exclusively related to ESCRT-dependent or independent category. Therefore, the extent of maturity of cerebellar cells in the derived spheroids does not seem to affect EV biogenesis. The EV biogenesis markers can be evaluated semi-quantitatively using Western blot as shown in our previous study and higher expression of EV biogenesis markers usually correlates with higher EV production or enriched exosome population^21,23^.

The EV isolation and nanoparticle tracking analysis (NTA) were then performed at the time points around day 14 (media collection at week 2–3) and day 35 (media collection at week 5–6) for the control and RCP conditions (Fig. 6C). NTA results showed that the mode size (120–157 nm) of the isolated EVs were comparable for the two conditions at the two time points. The mode size is usually a more accurate representation of the EV size as vesicle aggregation can affect the mean size. The particle concentrations (2.11–2.80 × 10^9^/mL for the control, and 2.49–3.85 × 10^9^/mL for the RCP condition) (Supplementary Table S8) and the protein content in the EVs were comparable for the control and RCP conditions at week 5–6, although the protein content at week 2–3 for the RCP condition was slightly lower. These observations in general were consistent with the results of gene expression.

### Zinc-induced neurotoxicity

**Figure 7:**
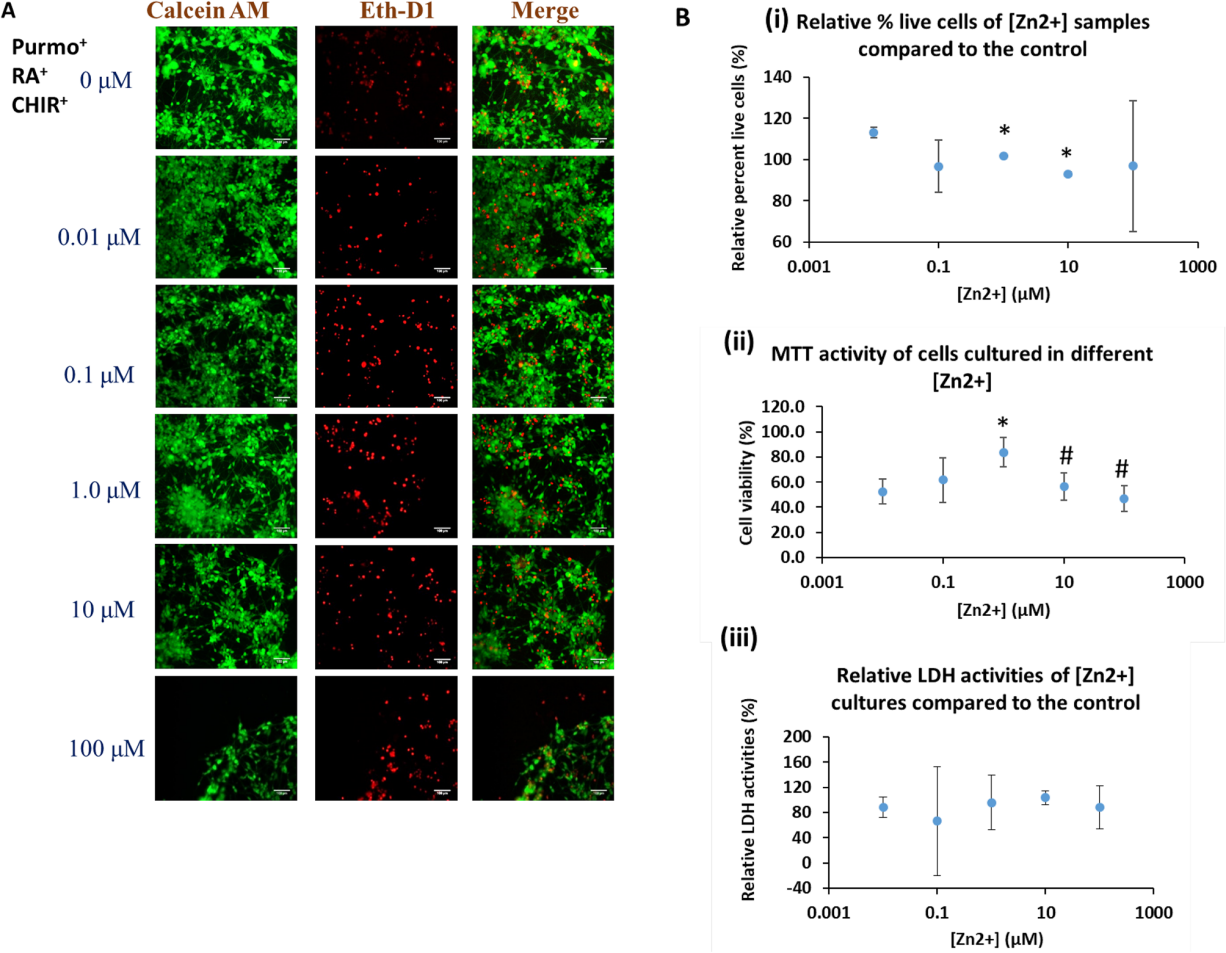
Zinc-induced neurotoxicity. (**A**) Images of LIVE/DEAD assay for the derived cerebellar cells exposed to Zinc at different concentrations. Scale bar: 25 µm. (**B**) (i) Relative percentage of live cells for the cells exposed to Zinc compared to the control. (ii) Relative MTT activity of cerebellar cells exposed to Zinc at different concentrations compared to the control. (iii) Relative LDH activity of cells exposed to Zinc compared to the control. n = 3. *p < 0.05 compared to the 0.01 µM condition. #*p* < 0.05 compared to the peak condition.

As a proof-of-concept study for the use of the derived cerebellar spheroids in neurotoxicity assessment, the derived day 35 cerebellar organoids were exposed to 0, 0.01, 0.1, 1.0, 10 and 100 µM Zinc supplemented culture media (Fig. 7). LIVE/DEAD assay was performed to examine the cell viability. From the images, majority of the cells exposed to the different zinc concentrations are live cells (Fig. 7A). Quantification of percentage of live cells over total cells showed the slight decrease in cell viability for 1.0 and 10 µM exposure compared to the control (Fig. 7B(i)). MTT assay was used to examine the cell metabolic activity. The MTT activity was found to reach the peak for 1.0 µM exposure and the decrease was observed for 10 and 100 µM exposure (Fig. 7B(ii)). LDH assay was performed for determining cytotoxicity. No significant differences were observed for different concentrations of zinc exposure (Fig. 7B(iii)). Taken together, mild zinc-induced neurotoxicity may exist when zinc exposure exceeds 1.0 µM.

## Discussion

In this study, the gene expression of different cerebellar markers along with metabolic pathway and EV biogenesis markers was determined using RT-PCR to characterize the cerebellar differentiation from hiPSCs by modulating RA, Wnt, and SHH pathways. Compared to forebrain cortical spheroids/organoids derived from hiPSCs, much less publications reported the derivation of hindbrain cerebellum spheroids/organoids^26^. Cerebellar differentiation from hPSCs has been reported recently in a PBS Vertical Wheel bioreactor based on the control condition (the Muguruma protocol 2015)^35^. The bioreactor culture accelerates the cerebellar differentiation, angiogenesis, and extracellular matrix based on the transcriptomic analysis^2,40^. The cerebellar maturation in the absence of co-culture was also reported^9^. For hindbrain patterning, retinoic acid and Wnt promotes the caudalization of the brain organoids and the activation of sonic hedgehog signaling induces ventralization of the brain tissues^41–43^. Our previous study showed the promoted cerebellar differentiation from hiPSCs by activating retinoid (using retinoic acid-R), Wnt (using CHIR99021-C), and Sonic Hedgehog (using purmorphamine-P) pathways^2^. The assessment was performed using common cerebellar markers including NEPH3, PTF1A, OLIG2, and MATH1.

Based on the human brain in cerebellum—The Human Protein Atlas, 12 of high score (RS > 20) general cerebellum genes and 14 of specific regional cerebellar layer genes were assessed in this study. The expression of 10 out of the 12 new cerebellar markers was promoted in the RCP-derived cerebellar spheroids compared to the control. The expression of 13 out of the 14 makers for specific cerebellar layers was promoted in the RCP-derived cerebellar spheroids. These results further confirm the effects of RCP on the patterning of cerebellar spheroids from hiPSCs and provides 23 novel markers in characterization of early cerebellar differentiation. To further examine the response of RA, Wnt, and SHH activation, the downstream genes need to be investigated in future (Supplementary Table S9–11).

Moreover, our results show that the cerebellar marker-enriched RCP-derived spheroids exhibit more active glycolytic activity and anaerobic metabolism, which may provide important biomarkers for cerebellar differentiation and process monitoring. While the shift toward glycolysis pathway for the spheroids with stronger cerebellar identity may not be cerebellar-specific, the results may provide general metabolic insights in monitoring neural differentiation from hiPSCs^44–46^. Glucose is the major fuel source for energy metabolism under normal and malignant conditions for brain cells. Undifferentiated hPSCs mainly use glycolysis to maintain pluripotency and the early stage differentiation induces the shift towards OXPHOS^10^. However, different brain cell types and polarization status may induce the metabolic pathway shift^47,48^. The metabolic status corresponding to the cerebellar differentiation and maturation status may need further investigation.

Our results demonstrate the minimal effect of cerebellar phenotype on the EV biogenesis in the RCP-derived cerebellar spheroids. Most of the examined ESCRT-dependent and independent EV biogenesis markers are comparable at day 35 for RCP-derived cerebellar spheroids compared to the control. The few downregulated genes *SMPD2* and *HRS* at day 35 were different from those downregulated at day 14. In general, the higher expression of EV biogenesis markers usually leads to the higher secretion of EVs. In this study, the differences in the EV mode size, EV numbers, and protein content were not statistically different around day 35, when the phenotype was mostly different for the control and RCP conditions. EV biogenesis could be affected by cytoskeleton alterations, hypoxia, pH, drug stimulation, and gene overexpression^18,49^. While the ability of the EV biogenesis of the RCP-derived cerebellar spheroids is retained, it is very likely that the protein and microRNA cargo of the secreted EVs is different due to the phenotypic difference of parent cells^50,51^. The EV cargo analysis usually requires the proteomics and microRNA profiling which has been shown for hiPSC-derived neural stem cells^52^.

Effect of Zinc on neural degeneration has been well recognized^34,53^. Histidine (His 6, His 13, and His 14) at the N-terminal domain of Aβ coordinates with Zn^2+^. Zinc is essential for brain function and tissue development. It is concentrated in the hippocampus and amygdala. Both Zn deficiency and Zn overload may affect cellular Zn homeostasis which is linked to neurodegeneration^34,53^. The predicted model for reactive oxygen species (ROS) level, cell viability, and cytotoxicity for zinc-induced toxicity in the derived cerebellar organoids is shown in Supplementary Fig. S3. Zn^2+^ ion is essential for cell development and scavenger protein production to reduce ROS level. Therefore, at low level of [Zn^2+^], it is hypothesized that cell viability increases until it reaches an optimal level; whereas, LDH and ROS levels decrease. When the Zn^2+^ level increases past the optimal concentration, it can induce ROS and harm the cells. Hence, cell viability may decrease fast and then slow down; whereas, LDH and ROS levels would increase fast and then are maintained at a certain value. Our results showed the mild zinc-induced neurotoxicity in the RCP-derived cerebellar spheroids when zinc exposure exceeds 1.0 µM. The effects of zinc might be different for different regions of human brain, which needs further investigation.

## Conclusions

This study identified some early cerebellar markers and regionally enriched genes in the RCP-derived cerebellar spheroids from hiPSCs. The results also provide the characterizations of metabolic pathways showing the elevated glycolysis in the more mature cerebellar spheroids. The EV biogenesis ability was retained in the derived cerebellar spheroids with different phenotype. As a proof-of-concept study, mild zinc-induced neurotoxicity was observed in the RCP-derived cerebellar spheroids when zinc exposure exceeds 1.0 µM. This study should advance the understanding of biomarkers during early cerebellar development for cerebellum organoid engineering and neurotoxicity study.

## Methods

### Undifferentiated hiPSC culture

Human foreskin fibroblasts were transfected with plasmid DNA encoding reprogramming factors octamer-binding transcription factor 4 (OCT4), NANOG, SRY-box transcription factor 2 (SOX2), and LIN28 to produce human iPSK3 cells (kindly provided by Dr. Stephen Duncan, Medical College of Wisconsin)^54,55^. The hiPSCs were maintained in mTeSR Plus serum free medium (StemCell Technologies, Inc., Vancouver, Canada) on growth factor-reduced Geltrex-coated surface (Life Technologies). The cells were passaged every seven days using Accutase and seeded at 1 × 10^6^ cells per well of six-well plate in the presence of rho-associated protein kinase (ROCK) inhibitor Y27632 (10 μM, Sigma) for the first 24 h^56,57^.

### Cerebellar spheroid differentiation from hiPSCs

Cerebellar differentiation was modified based on the published protocol^35,58^ as reported in our previous study^2^. Human iPSK3 cells were seeded at 3 × 10^5^ cells/well into ultra-low attachment 24-well plates (Corning Incorporated, Corning, NY) in differentiation medium composed of Dulbecco's Modified Eagle Medium/Nutrient Mixture F-12 (DMEM/F-12) with 2% B-27 serum-free supplements (Life Technologies). Y27632 (10 μM) and transforming growth factor (TGF)-β1 inhibitor SB431542 (10 μM, StemCell Technologies Inc.) were added for the first week of culture. On day 2, FGF2 (50 ng/mL, StemCell Technologies, Inc.) was added until the end of the first week. During the third week of culture, caudalization of neural rosettes in the derived aggregates was induced using FGF19 (100 ng/mL, Peprotech). Sequentially, the treatment of stromal cell-derived factor 1-α (SDF1A, 50 ng/mL, Peprotech) in the fifth week of culture was used to induce spheroid self-organization into the molecular layer, the Purkinje cell layer, and the granule cell layer. This procedure was used as a control condition.

The effects of casualization factor of retinoic acid (RA), wingless (Wnt) activator, and SHH activator were tested as shown in our previous study^2^. Briefly, during day 7–14, both RA (1.0 μM, Sigma) and Wnt activator CHIR99021 (CHIR, 10 μM, Sigma) were added to the spheroid culture of the control condition. In addition, during day 28–35, purmorphamine (PMR, 2 μM, Sigma) was used to activate the SHH pathway and promote ventralization of the spheroids. This condition was referred as RCP condition. On day 35, the gene and protein expression for cerebellar layer markers were characterized. As FGF8 has been reported to pattern rostral-caudal axis of during hPSC differentiation^36^, the effect of FGF8 treatment during day 7–21 was tested in comparison with RCP condition. For control and RCP groups, the spent media were collected and analyzed (n = 4) with a BioProfile Flex2 cell culture analyzer (Nova Biomedical) for metabolite analysis.

### Flow cytometry

The spheroids were trypsinized and different neural marker expression levels were quantified. Then, 1 × 10^6^ cells were fixed with 10% neutral formalin buffer (ThermoFisher) and washed with phosphate-buffered saline (PBS). The cells were permeabilized with 100% cold methanol for those with intracellular markers, blocked with blocking buffer (5% fetal bovine serum in PBS), and then incubated with different primary antibodies (Supplementary Table S1) followed by the corresponding secondary antibody Alexa Fluor 488 goat anti-Mouse IgG1. The cells were acquired using BD FACSCanto™ II flow cytometer (Becton Dickinson) and analyzed against isotype controls using FlowJo v10 software (https://www.bdbiosciences.com/en-us/products/software/flowjo-v10-software).

### Immunocytochemistry

For biomarker detection, the cells were fixed using 4% paraformaldehyde (PFA) and permeabilized using 0.2% Trixton-X 100. The samples were blocked with 5% FBS in PBS and stained with the primary antibodies (Supplementary Table S1), followed by the corresponding anti-species Alexa Fluoro antibodies, i.e., Alexa Fluor 488 goat anti-mouse IgG1 or Alexa Fluor 594 goat anti-Rabbit IgG (Life technologies). Both primary and secondary antibody dilutions were made based on the manufacturer’s recommendations and prepared in staining buffer (2% FBS in PBS). Then the nuclei were counterstained with Hoechst 33342 (blue), and pictures were taken for blue, green, and red colors to detect the markers and their cellular locations under a fluorescent microscope (Olympus IX70, Melville, NY).

### Extracellular vesicle isolation

Extracellular vesicles (EVs) were isolated from the conditioned media (around week 2–3 and week 5–6) by polyethylene glycol (PEG) precipitation and ultracentrifugation following our previous publications^21,23^. Briefly, cells were cultured in DMEM-F12 with serum-free B27 and the conditioned media were collected every 48–72 h. The conditioned media were then differential centrifuged (500*g* for 5 min; 2000*g* for 10 min; 10,000*g* for 30 min) to remove larger debris, apoptotic body and microvesicles. Supernatants were then mixed with PEG solution (24% wt/vol PEG, 1.5 M NaCl) at a 1:1 volume and incubated at 4 °C overnight. The next day, the mixed solutions were centrifuged at 3214*g* for 1 h to obtain crude EVs. The pellets were resuspended in PBS and then ultracentrifuged at 100,000*g* for 70 min. Purified EVs were resuspended in 100 µL particle-free PBS and stored in − 80 °C for further use.

### Nanoparticle tracking analysis (NTA)

NTA was performed on the isolated EV samples in triplicate to determine size distribution and particle concentration. NTA was performed on a Nanosight LM10-HS instrument (Malvern Instruments, Malvern, UK) configured with a blue (488 nm) laser and CMOS camera^59^. For each replicate, three videos of 60 s were acquired with camera shutter speed fixed at 30.00 ms. To ensure accurate and consistent detection of small particles, camera level was set to 13, and detection threshold was maintained at 5. The laser chamber was cleaned thoroughly with particle-free water between each sample reading. The collected videos were analyzed using NTA3.4 software (NanoSight NTA software v3.4 in the instrument) to obtain the mode and mean size distribution, as well as the concentration of particles per mL of solution.

### Reverse transcription-polymerase chain reaction (RT-PCR) analysis

Total RNA was isolated from the neural spheroids using the RNeasy Mini Kit (Qiagen, Valencia, CA) following the manufacturer’s protocol followed by the treatment of DNA-Free RNA Kit (Zymo, Irvine, CA). Reverse transcription was carried out using 2 μg of total RNA, anchored oligo-dT primers (Operon, Huntsville, AL), and Superscript III (Invitrogen, Carlsbad, CA) (according to the protocol of the manufacturer). Primers specific for target genes (Supplementary Table S2–S10) were designed using the Primer-BLAST (NCBI), and the melting temperature was checked using NetPrimer Analysis (PREMIER Biosoft). The gene β-actin was used as an endogenous control for normalization of expression levels. Real-time RT-PCR reactions were performed on an ABI7500 instrument (Applied Biosystems, Foster City, CA) using SYBR1 Green PCR Master Mix (Applied Biosystems). The amplification reactions were performed as following: 2 min at 50 °C, 10 min at 95 °C, and 40 cycles of 95 °C for 15 s and 55 °C for 30 s, and 68 °C for 30 s. Fold variation in gene expression was quantified by means of the comparative 2^−ΔΔCT^ method based on the comparison of expression of the target genes (normalized to the endogenous control β-actin) among different conditions.

### Western blot assay

Cells were lysed in radio-immunoprecipitation assay (RIPA) buffer (150 mM sodium chloride, 1.0% Triton X-100, 0.5% sodium deoxycholate, 0.1% sodium dodecyl sulfate, 50 mM Tris, pH 8, 2 µg/mL Aprotinin, 5 µg/mL Leupeptin, 5 µg/mL Antipain, 1 mM PMSF protease inhibitor), and homogenized by sonification using a Sonic Dismembrator 100 (Fisher Scientific, Hampton, NJ). Samples were then digested for 20 min on ice, and spun down at 14,000 rpm for 20 min. The supernatant was collected and a Bradford assay was carried out to determine the protein concentration. Protein lysate concentration was normalized, and 20 µg of each sample was denatured at 95 °C in 2 × Laemmli Sample buffer. Proteins were separated by 15% BIS–Tris-SDS gels and transferred onto a nitrocellulose membrane (Bio-rad, Hercules, CA). For the detection of non-phosphorylated proteins, the membranes were blocked for 30 min in 3% skim milk (w/v) in Tris-buffered saline (10 mM Tris–HCl, pH 7.5, and 150 mM NaCl) with 0.1% Tween 20 (v/v) (TBST), or in 3% bovine serum albumin in TBST. Membranes were incubated overnight in the presence of the primary antibody diluted in the corresponding blocking buffer at 4 °C. Afterward, the membranes were washed four times for 10 min each with TBST and then incubated with an IR secondary (LI-COR, Lincoln, NE) at 1:10,000 for 180 min at room temperature. Blots were washed another four times for 10 min each with TBST and processed using the LI-COR Odyssey (LI-COR). Images were analyzed using ImageJ 1.46r software (https://imagej.nih.gov/ij/, National Institutes of Health, USA) for band density, and the band density of proteins of interest was normalized to the band density of endogenous control β-actin.

### Zinc-induced neurotoxicity

The derived day 35 cerebellar organoids were replated onto the Matrigel-coated 24-well plates and exposed to 0, 0.01, 0.1, 1.0, 10 and 100 µM Zinc supplemented culture media for three days. The supplementation of ZnSO4·7H2O (Sigma) to the media was performed to generate Zinc ion at the target concentrations. The cells were then examined by LIVE/DEAD assay and MTT assay. The spent media were saved for lactate dehydrogenase assay.

### (4,5-dimethylthiazol-2-yl)-2,5-diphenyltetrazolium bromide (MTT) assay

The day 35 replated cerebellar spheroids exposed to different concentration of zinc were incubated with a 0.5 mg/mL MTT (Sigma) solution for an hour at 37 °C. The media and MTT were removed. The formazan crystals were dissolved in dimethyl sulfoxide and centrifuged at 800*g* for 5 min. The absorbance of the supernatants was measured at 490 nm on a microplate reader (BioRad Laboratories, Hercules, CA).

### LIVE/DEAD staining

The cells were evaluated for viability using the LIVE/DEAD staining kit (Molecular Probes) according to the manufacturer’s protocol. The day 35 cerebellar spheroids were replated onto Matrigel-coated surface and exposed to Zinc at different concentrations. After Zinc treatment, the cells were washed with PBS and then incubated in DMEM-F12 containing 3–10 μM calcein-AM (green) and 8 μM ethidium homodimer I (red) for 20 min at room temperature and protected from light. The images were taken for the spheroid outgrowth under a fluorescent microscope (Olympus IX70, Melville, NY). Image analysis was performed using ImageJ 1.46r software (https://imagej.nih.gov/ij/, National Institutes of Health, USA) on at least three images for each condition. The viability was analyzed and calculated as the percentage of green intensity over total intensity (including both green cells and red cells).

### Lactate dehydrogenase (LDH) activity assay

The cytotoxicity was assessed using LDH activity assay kit (Sigma, MAK066). Briefly, a total volume of 100 μL of spent medium of zinc-treated cerebellar spheroids and LDH reaction mixture were mixed. The initial absorbance at 450 nm was measured using a microplate reader (Bio-Rad iMark). The mixture was incubated at 37 °C and taken measurement every 5 min. The LDH activity was calculated through the subtraction of final and initial measurements in comparison to the standard curve.

### Statistical analysis

The representative experiments were presented, and the results were expressed as [mean ± standard deviation]. To assess the statistical significance, one-way ANOVA or student’s t-test followed by Fisher’s LSD post hoc tests were performed. A p-value < 0.05 was considered statistically significant.

## Supplementary Information

Supplementary Information.
