# Supplementary material for: Phenotypic, metabolic, and biogenesis properties of human stem cell-derived cerebellar spheroids
Source: Sci Rep. 2022 Jul 27;12:12880. doi: 10.1038/s41598-022-16970-1 (PMC9329474; doi:10.1038/s41598-022-16970-1)

**Supplementary Information**

**Phenotypic, Metabolic, and Biogenesis Properties of Human Stem Cell-derived Cerebellar Spheroids**

Timothy Hua^1,#^, Chang Liu^2,#^, Sonia Kiran ^1^, Kelly Gray ^3^, Sunghoon Jung ^3^,

David G. Meckes Jr. ^4^, Yan Li^2, 5*^, Qing-Xiang Amy Sang^1, 5 *^

^1^Department of Chemistry and Biochemistry, Florida State University, Tallahassee, Florida, USA

^2^Department of Chemical and Biomedical Engineering, FAMU-FSU College of Engineering, Florida State University, Tallahassee, Florida, USA

^3^PBS Biotech Inc., Camarillo, California, USA

^4^Department of Biomedical Sciences, College of Medicine, Florida State University, Tallahassee, Florida, USA

^5^Institute of Molecular Biophysics, Florida State University, Tallahassee, Florida, USA

**# co-first authors**

**Supplementary Table S1. A list of antibodies.**

| **Cells** | **Primary Antibody** | **Origin/ Isotype** | **Supplier/ Cat#** | **Dilution** |
| --- | --- | --- | --- | --- |
| General neuronal marker | Beta-tubulin III | Mouse IgG1 | Millipore, MAB1637 | 1:200 |
| Cerebellar markers | NEPH3 | Mouse IgG1 | Santa Cruz, sc-515104 | 1:50  1:1000 (Western) |
|  | PTF1A | Mouse IgG1 | Santa Cruz, sc-393011 | 1:50 |
|  | OLIG2 | Mouse IgG1 | Santa Cruz, sc-515947 | 1:100  1:1000 (Western) |
|  | MATH1 | Mouse IgG1 | Santa Cruz, sc-136173 | 1:100 |
|  | FAT2 | Mouse IgG1 | Santa Cruz, sc- sc-59985 | 1:100 |
|  | GABRA6 | Mouse IgG1 | Santa Cruz, sc-376282 | 1:100 |
|  | HOMER3 | Mouse IgG1 | Santa Cruz, sc-365254 | 1:100 |
|  | KCNIP4 | Mouse IgG1 | Santa Cruz, sc-373732 | 1:100 |
| Secondary | Alexa 488, goat anti-mouse IgG1 | - | Life Technologies,  A-21121 | 1:200 |

**Supplementary Table S2. Twelve genes with highest RS-score in the human cerebellum.**


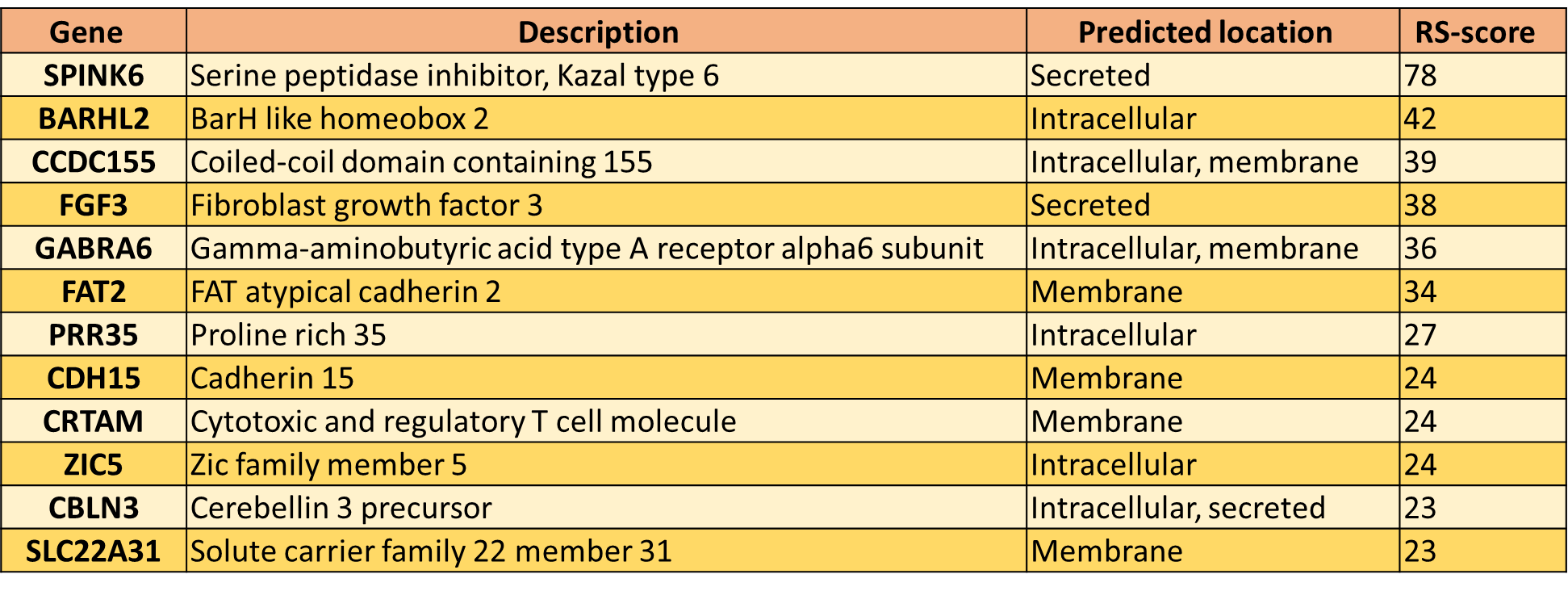


**Supplementary Table S3. The primer pairs for the twelve genes with highest RS-score in the human cerebellum.**


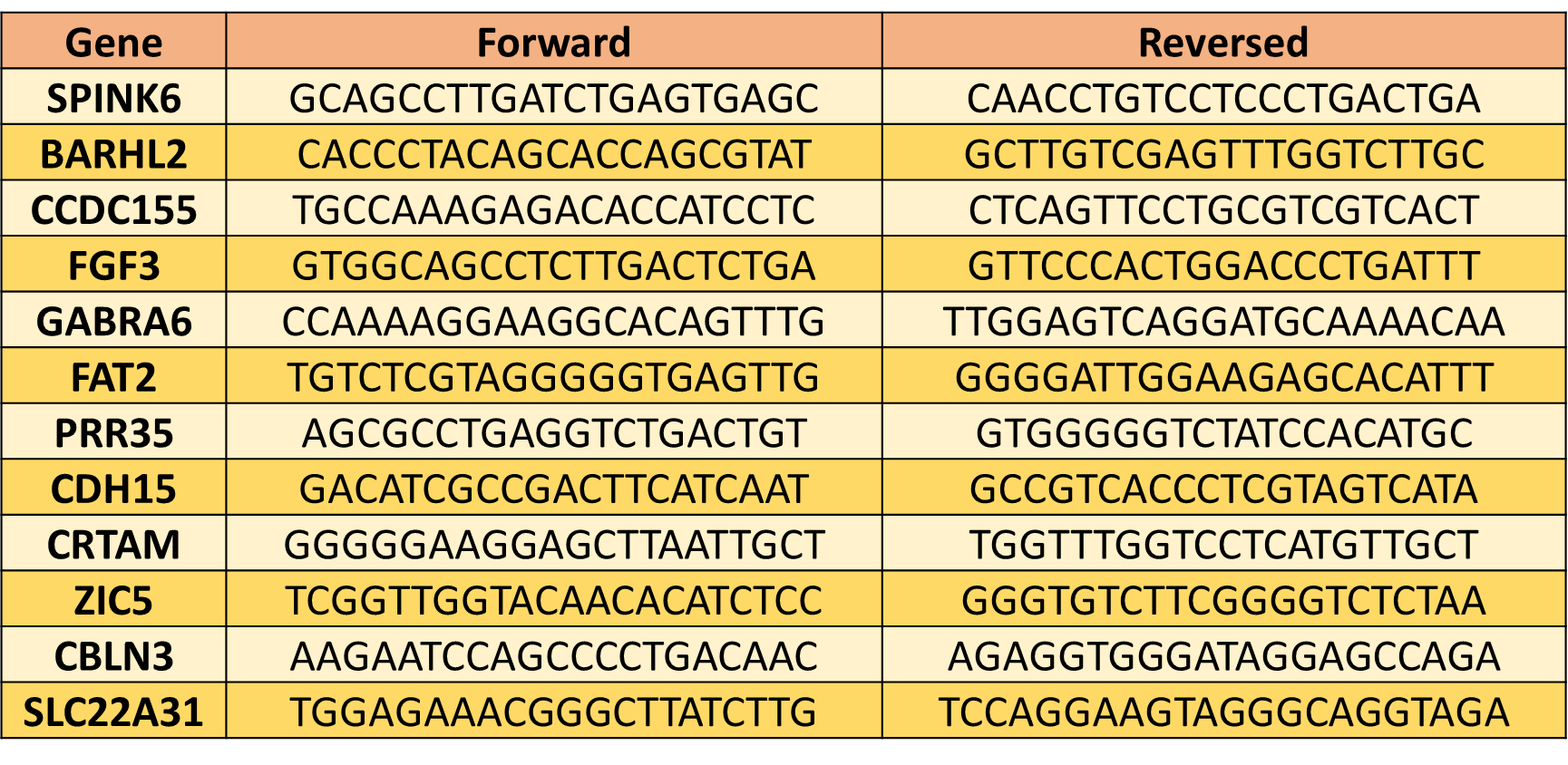


**Supplementary Table S4. The specific genes for the molecular layer, the Purkinje cell layer, the granule cell layer, and the Bergmann glial cells of the human cerebellum.**


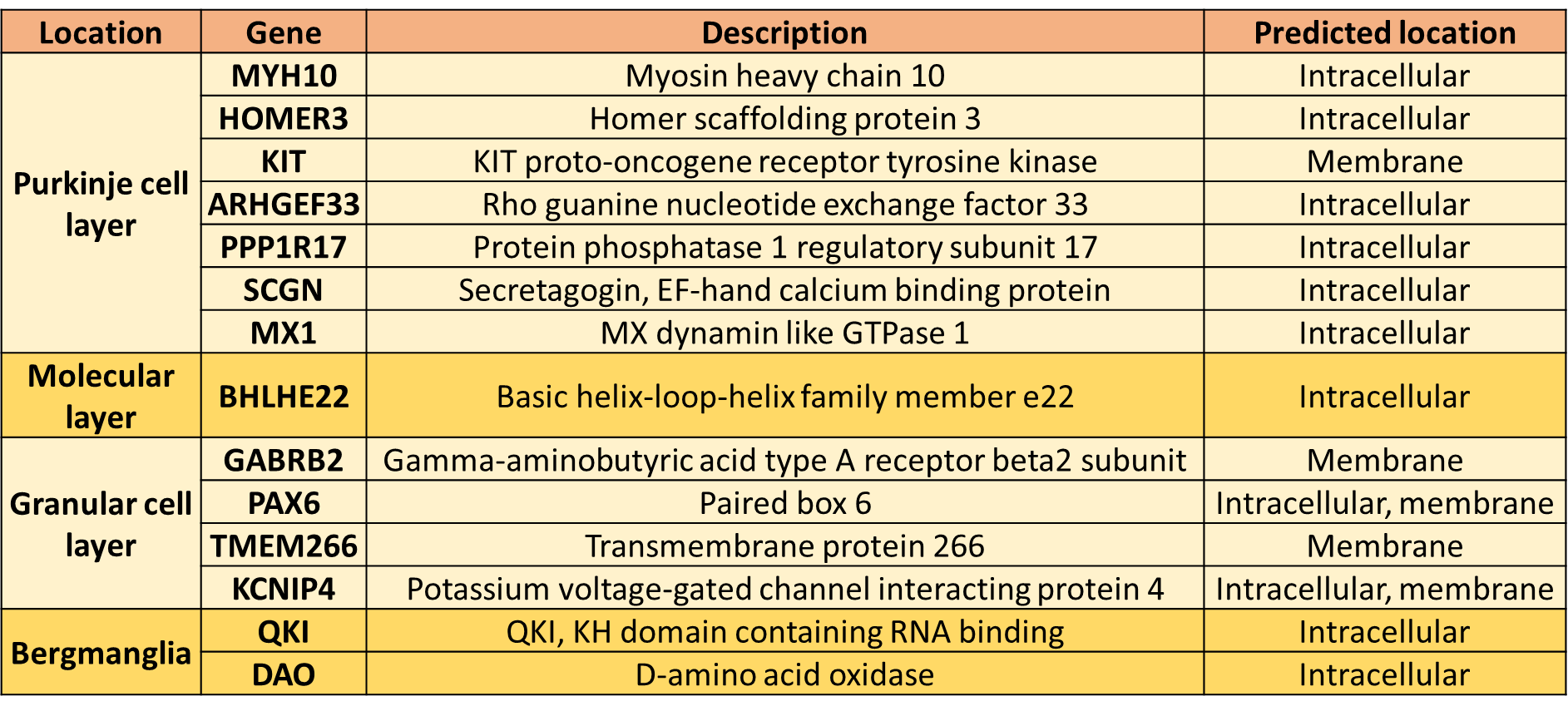


**Supplementary Table S5. The primer pairs for the specific genes for the molecular layer, the Purkinje cell layer, the granule cell layer, and the Bergmann glial cells of the human cerebellum.**


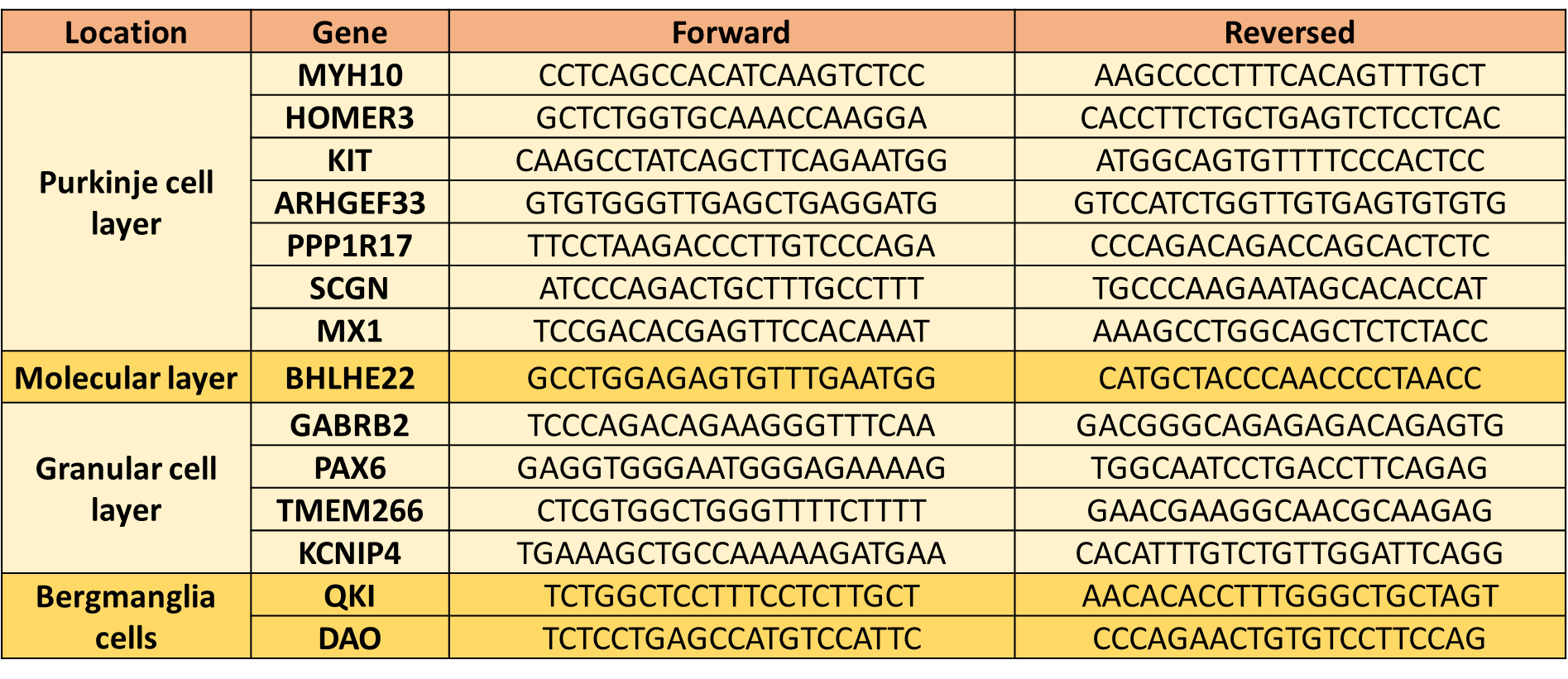


**Supplementary Table S6. Primer pairs for genes related to metabolic pathways.**

| **P number** | **Name** | **Name in database** | **Primers** |
| --- | --- | --- | --- |
|  | ACTB | Bactin F | GTACTCCGTGTGGATCGGCG |
|  |  | Bactin R | AAGCATTTGCGGTGGACGATGG |
|  | GAPDH | GAPDH-F | TCACTGCCACCCAGAAGACTG |
|  |  | GAPDH-R | GGATGACCTTGCCCACAGC |
| 1 | PDK1 | PDK1-F1 | AAACAGGGGAGCTTTGTCTGG |
|  |  | PDK1-R1 | CTGCCCATTCACATCCCTCTA |
| 2 | HK2 | HK2_F1 | TGGTGTAGCTCCTCTGCTGCT |
|  |  | HK2_R1 | TGTGGGCACCCTTTAGTGAAC |
| 3 | PKM2 | PKM2-F1 | AAAAATGGATGCCCAGAGGAC |
|  |  | PKM2-R1 | GAGTCGGCTTCAATGGAACAA |
| 4 | LDHA | LDHA-F1 | CCTTGAGCCAGGTGGATGTTT |
|  |  | LDHA-R1 | CACTGGATCCCAGGATGTGAC |
| 5 | G6PD | GL6PD-F1(G6PD) | CTACCCGAGCCCAGCTACATT |
|  |  | GL6PD-R1 | TTCTGTTGGGCTGGAGTGAGT |
| 6 | 6PGD | 6PGLD-F1(6PGD) | CCATGCCCTGTTTTACCACTG |
|  |  | 6PGLD-R1 | AGGTGTGAGCCCCGAAGTAAT |
| 7 | TALDO1 | TALDO F1 | CTGTCATCAACCTGGGAAGGA |
|  |  | TALDO R1 | GGGCGAAGGAGAAGAGTAACG |
| 8 | TKTL1 | TKTL1 F1 | ACCTTGGGATTCTGTGTGCTG |

**Supplementary Table S7. Primer pairs for extracellular vesicle biogenesis.**

| **P number** | **Name** | **Name in database** | **Primers** |
| --- | --- | --- | --- |
|  | ACTB | Bactin F | GTACTCCGTGTGGATCGGCG |
|  |  | Bactin R | AAGCATTTGCGGTGGACGATGG |
|  | GAPDH | GAPDH-F | TCACTGCCACCCAGAAGACTG |
|  |  | GAPDH-R | GGATGACCTTGCCCACAGC |
| 1 | SMPD2-1 | SMPD2-F1 | GCCTGGGAGACTTTCTGAACC |
|  |  | SMPD2-R1 | AAGTGGTGTGCAGCTGGGTAG |
| 2 | SRSF5(hrs)-1 | SRSF5-F1 | CTTCTCGGATCGAGGCTTCTT |
|  |  | SRSF5-R1 | TCGAATCAACTGCGCTCATTA |
| 3 | TSG101 | TSG101 F | CACCTGGTGGTCCATATCCTG |
|  |  | TSG101 R | GATGGTGTCCTCGCTGATTGT |
| 4 | STAM1 | STAM1-F1 | CACTGGATTTTTGGGTTGCTC |
|  |  | STAM1-R1 | GTGGAAAACATTTTTCGCATGA |
| 5 | PDCD61P (ALIX) | PDCD61P F | TAAGTGCATCTGAGGGCCAAA |
|  |  | PDCD61P R | GGGGCCTCCTTTCCTAGTTTC |
|  | PDCD61PI4 (ALIXI4) | PDCD61Pi4 F | TTGGCTAATCAGGCTGCAGAT |
|  |  | PDCD61Pi4 R | TCACATGCAAAGTAAGCAAGTGTT |
| 6 | MITF-1 | MITF-F1 | GAATTGGTGATGGGTGATGGA |
|  |  | MITF-R1 | TGCATGGGAACTATGCAGTTG |
| 7 | RAB27A | RAB27A F | GCATGTTTCAGTTTTCAAGAACCA |
|  |  | RAB27A R | AAAGGTGGCTTTTGTGTGTGC |
| 8 | CD63 | CD63 F | ACAACCACACTGCTTCGATCC |
|  |  | CD63 R | GACTCGGTTCTTCGACATGGA |

**Supplementary Figure S8. Extracellular vesicle particle concentration determined by NTA.**

| **Conditions** | **CRTL**  **Week 2-3** | **CRTL**  **Week 5-6** | **RCP**  **Week 2-3** | **RCP**  **week 5-6** |
| --- | --- | --- | --- | --- |
| **Run 1 (x10^9^/mL spent media)** | **2.80** | **8.26*** | **3.43** | **3.85** |
| **Run 2**  **(x10^9^/mL spent media)** | **2.11** | **2.40** | **5.24** | **2.49** |

*This isolation is unusual and has protein degradation.

**Supplementary Table S9. Primer pairs for retinoic acid pathway components.**


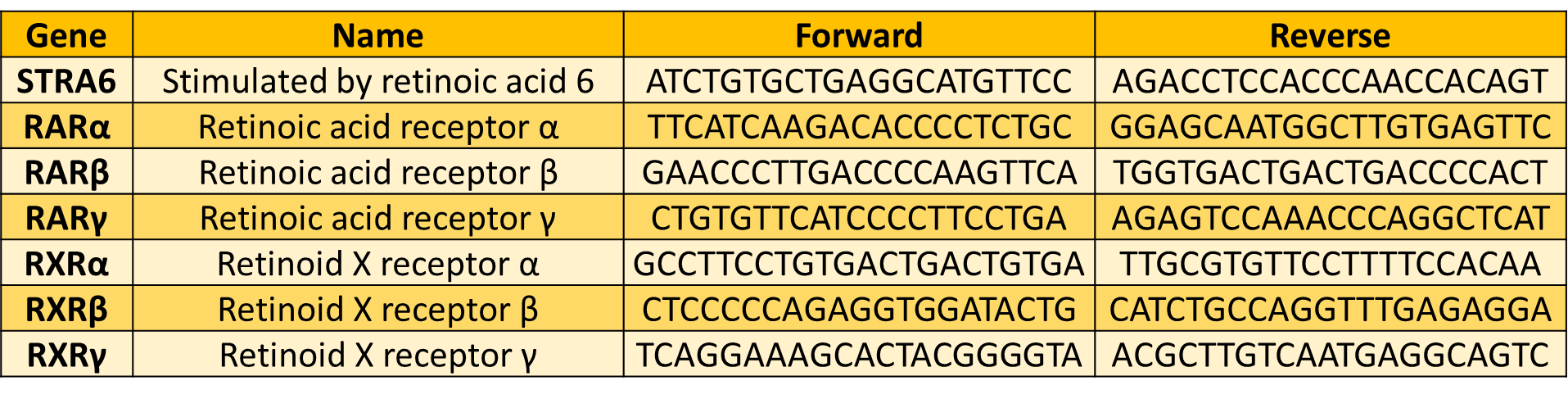


**Supplementary Table S10. Primer pairs for Wnt pathway components.**


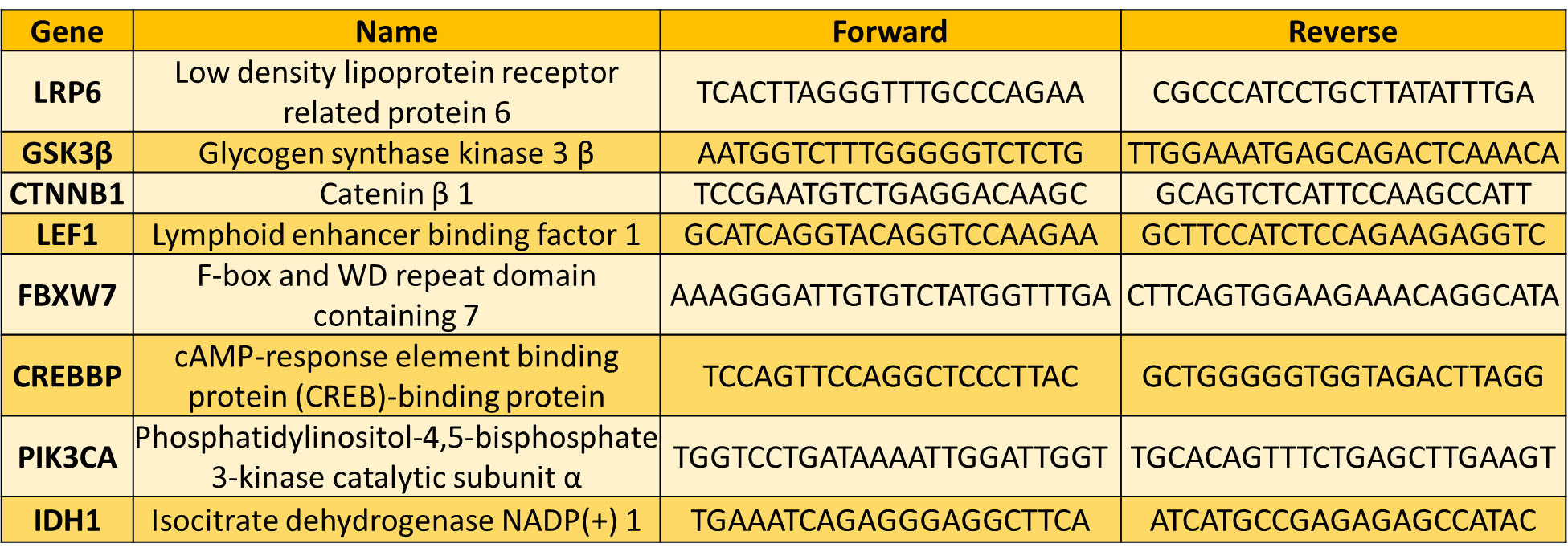


**Supplementary Table S11. Primer pairs for SHH pathway components.**


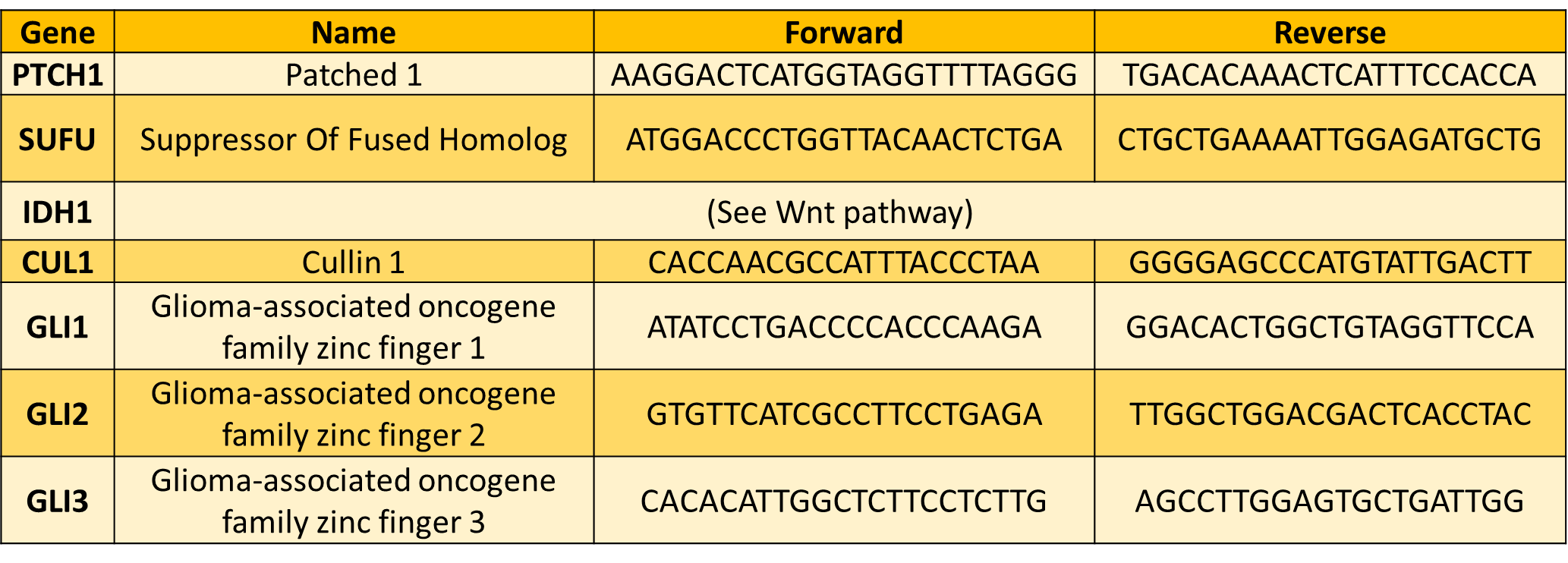


**Supplementary Figure S1. Western blot analysis of cerebellar markers at day 35.** RCP, RC, and CP are the three treated conditions. R: retinoid acid; C: CHIR99021, P: Purmorphamine. (A) Western blot band; The blots were cut prior to hybridisation with antibodies. (B) Quantification of band density (normalized to β-actin). * *p*<0.05. RC+ group showed higher expression probably due to the uneven loading. Adding P to the PC group should not reduce the expression level.


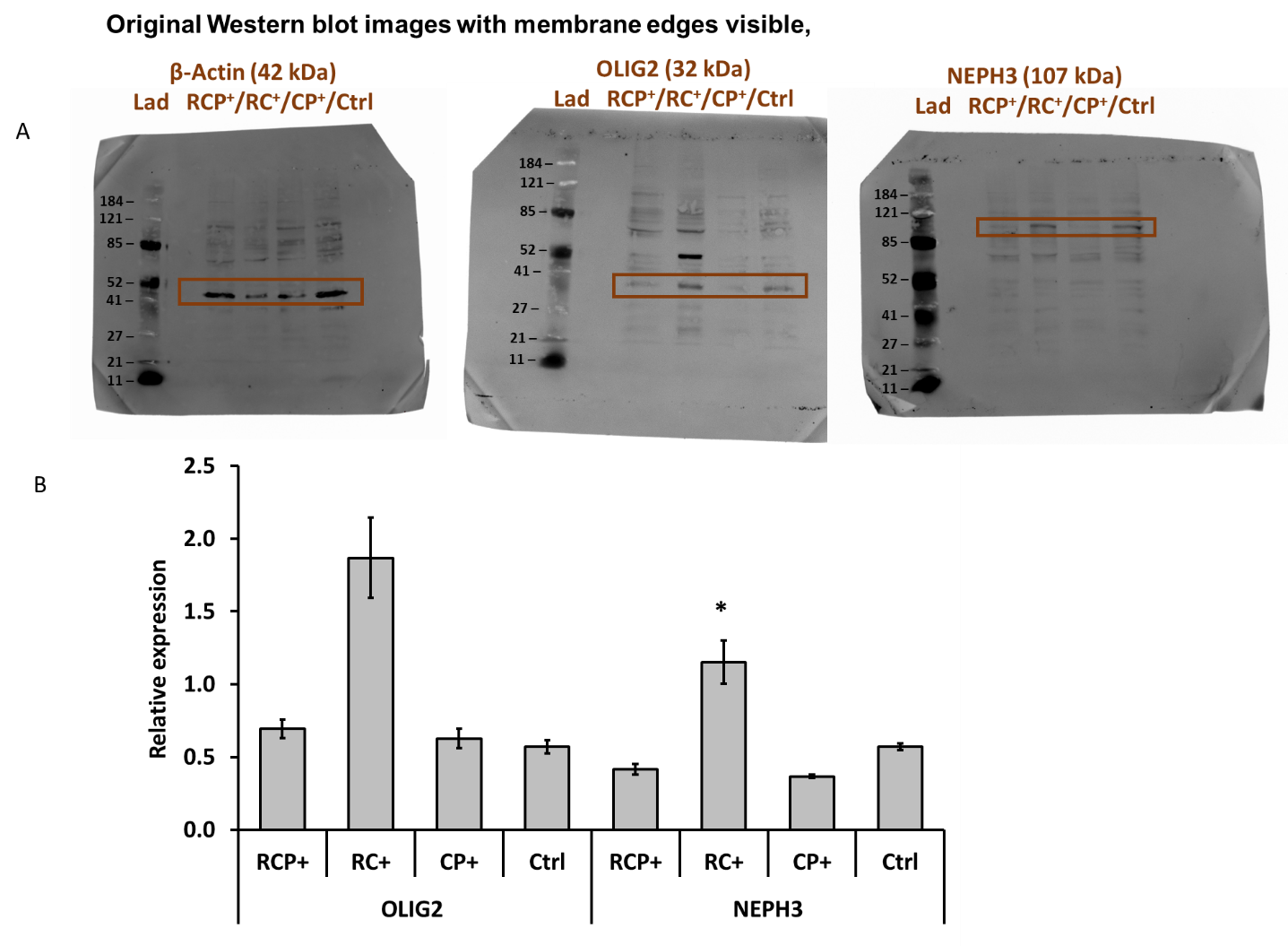


**Supplementary Figure S2. Additional metabolite analysis for cerebellar differentiation.** (A) Glutamate consumption and (B) ammonia generation. Expression levels of (C) Na+, (D) K+; and (E) Ca2+. * indicates *p*<0.05.

**
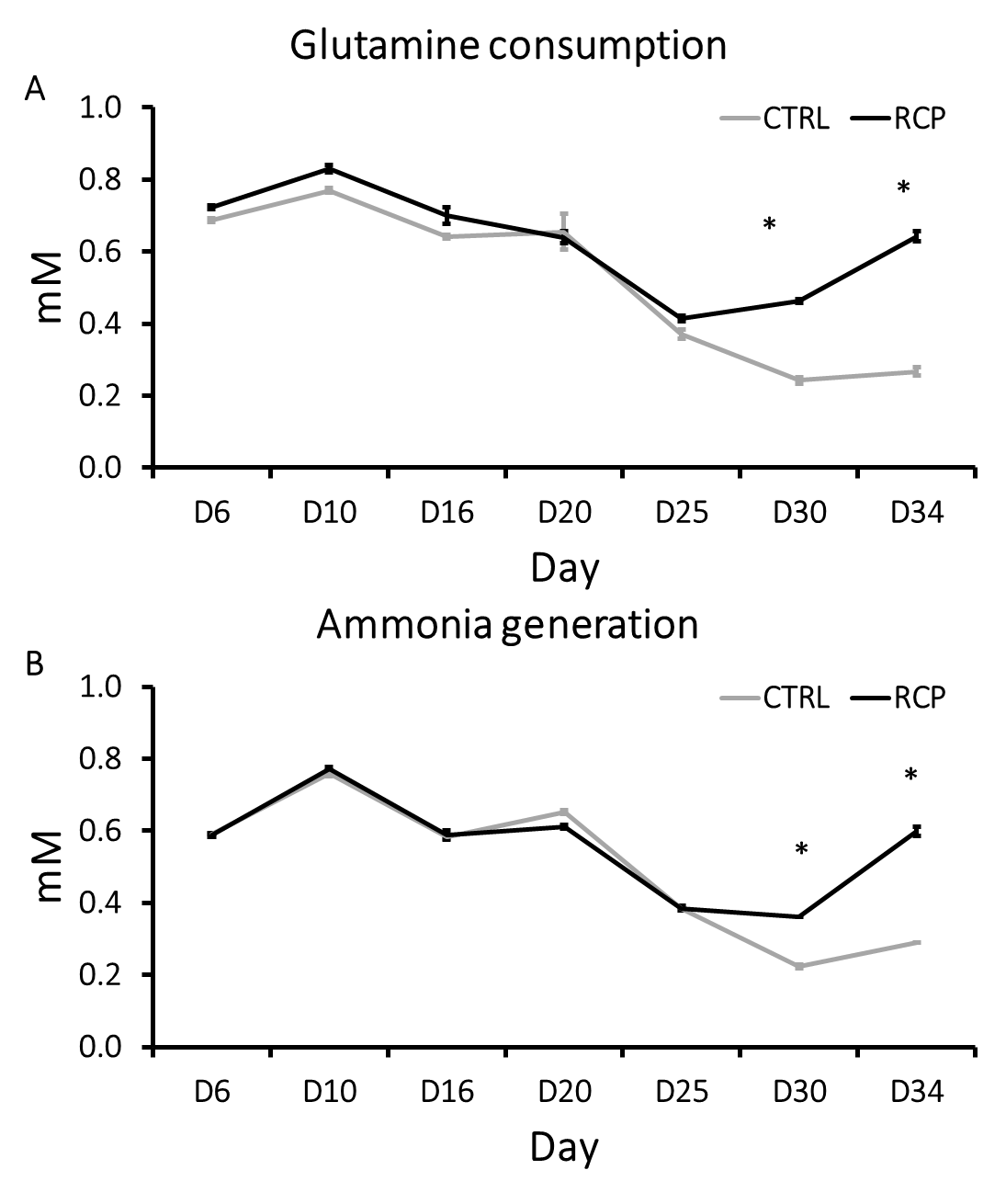
**

**
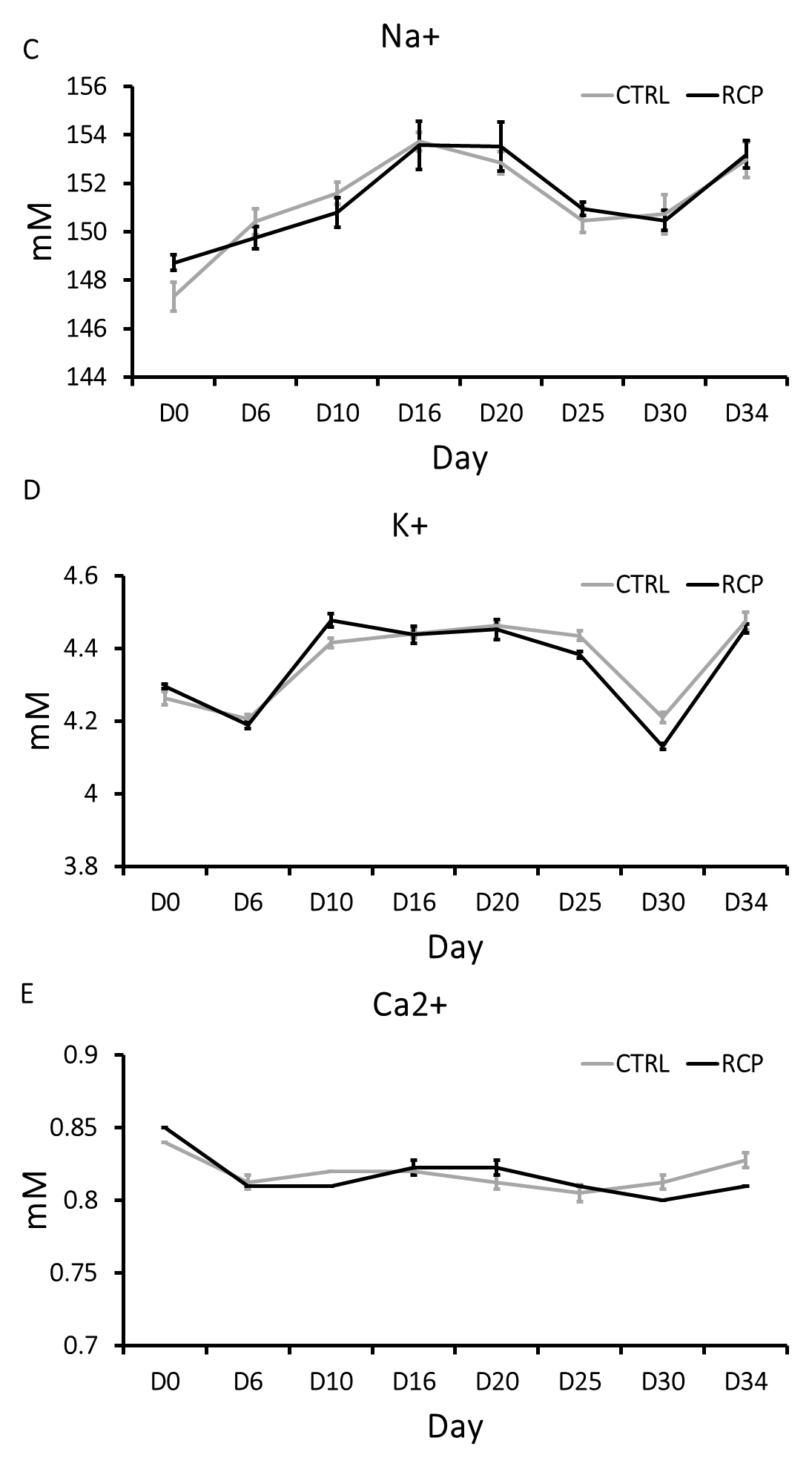
**

**Supplementary Figure S3. The predicted model for reactive oxygen species (ROS) level, cell viability, and cytotoxicity for zinc-induced toxicity in the derived cerebellar organoids.**


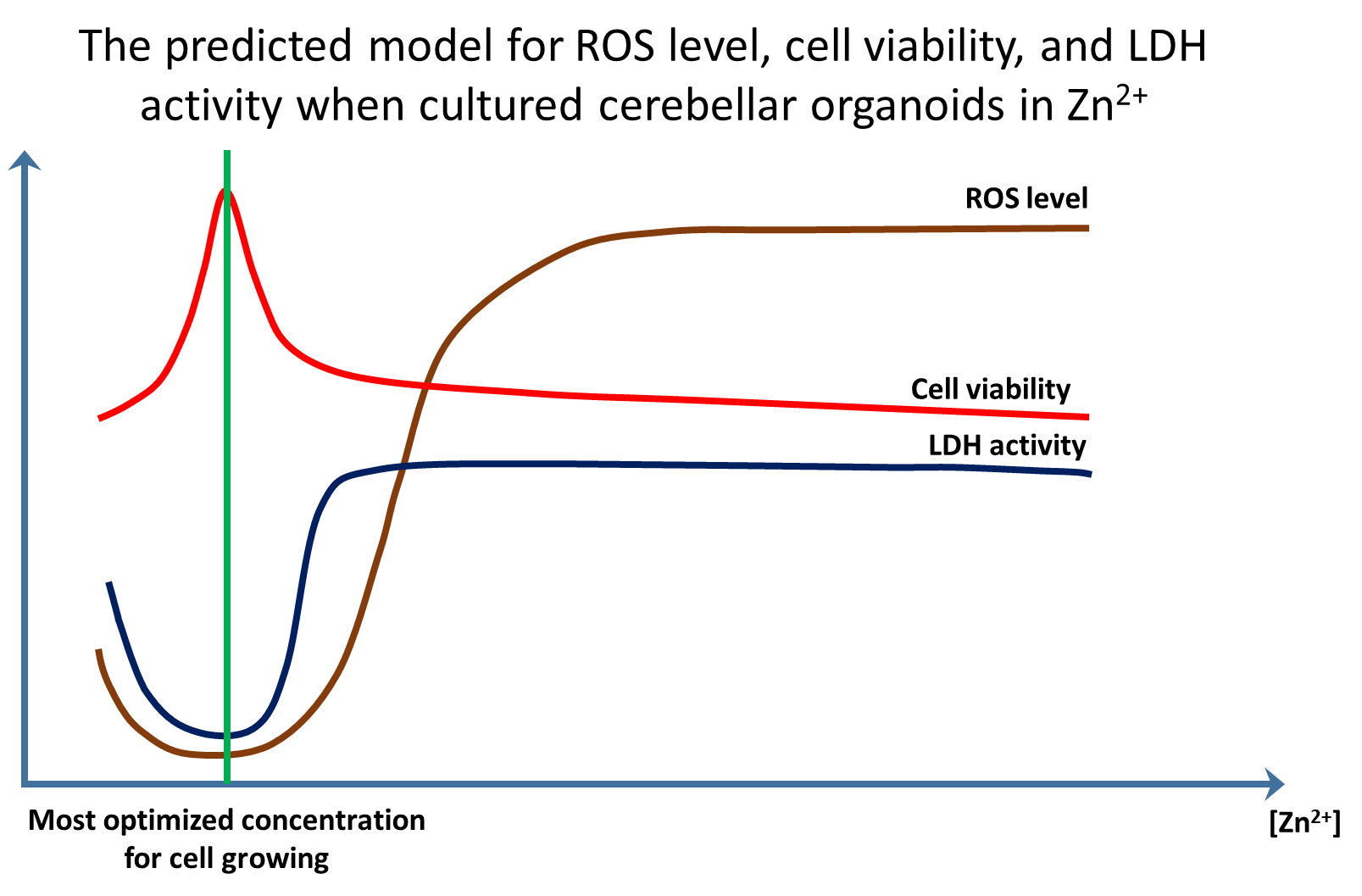

Supplement: Supplementary file 1 — Supplementary Information. [file 41598_2022_16970_MOESM1_ESM.docx]
